# Four nudivirus core genes present in the genome of *Venturia canescens* are required for virus-like particle formation and prevention of encapsulation of parasitoid wasp eggs

**DOI:** 10.1128/jvi.01305-25

**Published:** 2025-11-20

**Authors:** Meng Mao, Corinne M. Stouthamer, Ange Lorenzi, Michael R. Strand, Gaelen R. Burke

**Affiliations:** 1 Department of Entomology, University of Georgia 166833, Athens, Georgia, USA; 2 DGIMI, University of Montpellier, INRAE, Montpellier, France; Wageningen University & Research, Wageningen, Netherlands

**Keywords:** parasitoid, nudivirus, endogenous virus element, virus-like particle

## Abstract

*Venturia canescens* is a parasitoid wasp that harbors a domesticated endogenous virus (DEV) and parasitizes host insects like *Ephestia kuehniella*. The *V. canescens* DEV evolved from an alphanudivirus and produces virus-like particles (VLPs) in females that protect wasp eggs from a host immune defense called encapsulation. In contrast, very few DEV genes required for VLP formation and function have been identified. In this study, we characterized five *V. canescens* DEV genes of unknown function that all nudiviruses encode. Three of these genes are single copy (*OrNVorf18-like*, *OrNVorf61-like*, and *OrNVorf76-like*), while *OrNVorf41-like* has expanded into a six-member family and *OrNVorf47-like* has expanded into a three-member family. Sequence analysis indicated all of these genes retain essential motifs present in nudivirus homologs, while transmission electron microscopy (TEM) studies characterized the timing of VLP formation during the wasp pupal stage. RNA interference (RNAi) assays identified *OrNVorf18-like*, *OrNVorf61-like*, *OrNVorf41-like-1,* and *OrNVorf41-like-2* as genes that are required for normal VLP formation. Knockdown of *OrNVorf47-like* family members did not affect VLP formation but did disable binding of VLPs to *V. canescens* eggs and protection against encapsulation. Disabled formation of VLPs in response to RNAi knockdown of *OrNVorf18-like*, *OrNVorf61-like*, *OrNVorf41-like-1,* and *OrNVorf41-like-2* also resulted in wasp eggs being encapsulated. In contrast, knockdown of *OrNVorf76-like* had no effect on VLP assembly, egg binding, or encapsulation. Altogether, reported results significantly advance our understanding of *V. canescens* VLP (VcVLP) formation and function.

**IMPORTANCE:**

Understanding how *V. canescens* coopted an alphanudivirus to produce VcVLPs is of interest to the study of virus evolution. Our results show that three nudivirus core genes have essential functions in VcVLP formation, while one is essential for the novel function of binding to wasp eggs and protection from encapsulation, which is the most important immune defense of insects against parasitoids.

## INTRODUCTION

Parasitoid wasps (order Hymenoptera) are likely the most diverse animal group on Earth, with estimates of more than a million species (1, 2). Most are free-living insects as adults that parasitize other insects by laying eggs on or in their bodies (3). Viruses in the family *Nudiviridae* have large, circular double-stranded (ds) DNA genomes, specifically infect insects or other arthropods, and replicate in the nuclei of infected cells where they produce virions consisting of an envelope and one or more capsids (4). Different nudiviruses have integrated into the germline of certain parasitoid lineages and become Domesticated Endogenous Viruses (DEVs) (5). Each retains many nudivirus genes, which are specifically expressed in ovary calyx cells of females and produce either DNA-containing virions or virus-like particles (VLPs) (6–9). DEV-produced virions or VLPs cannot replicate, but wasps maintain the ability to produce them because all genome components necessary to do so are vertically transmitted to offspring. Each wasp lineage has also convergently evolved to use DEV-produced virions or VLPs to transfer genes, proteins, or other factors to host insects, which promote the successful development of offspring.

The most studied of these DEVs are bracoviruses (BVs), which evolved from a betanudivirus-like ancestor that integrated ~100 million years ago into a parasitoid in the family Braconidae (6, 10). Subsequent speciation has resulted in thousands of braconids that produce BV virions, which consist of an envelope, one or more capsids, and DNAs that are amplified from the wasp genome and packaged into capsids (11, 12). In contrast, all of the genes with functions in producing virions remain integrated in the genomes of wasps. Many of the genes with virion-producing functions have been characterized using RNA interference (RNAi) assays in conjunction with transmission electron microscopy (TEM) and other methods (13–15). The BV virions that females inject into hosts when laying eggs infect different cell types, which is followed by the expression of virulence genes that reside on the DNAs that are packaged into capsids (16, 17). Several of these virulence genes have also been characterized and shown to alter host immune defenses, growth, and behavior (12, 18–20).

Less studied are ichneumonid wasps in the genus *Venturia,* where species like *V. canescens* contain a DEV that evolved from an alphanudivirus similar to Oryctes rhinoceros nudivirus (OrNV) (8). The *V. canescens* DEV produces 130 nm VLPs that consist of a spheroidal envelope containing proteins but no capsid or nucleic acid (21, 22). *V. canescens* VLPs (VcVLPs) assemble in calyx cell nuclei and accumulate in the calyx lumen (22, 23). Proteomic analysis of VcVLPs identifies many nudivirus gene products (8), while prior findings also show that VcVLPs associate with *V. canescens* eggs when passaging through the calyx lumen before storage in the lateral oviducts (22). *V. canescens* females parasitize larval stage moths like *Ephestia kuehniella* by usually laying one egg per host. Experiments indicate that the association of VcVLPs with eggs is also involved in protection from host hemocytes, which otherwise mount a fatal defense response called encapsulation (23, 24).

All sequenced viruses in the *Nudiviridae* encode 32 core genes, of which 21 are shared with viruses in the *Baculoviridae* that also have large, circular dsDNA genomes, infect insects or other arthropods, and replicate in host cell nuclei (25). None of these shared core genes have been experimentally studied in nudiviruses, whereas all have been characterized in model baculoviruses (26). Genome sequencing of *V. canescens* followed by a chromosome-level assembly of the genome identifies 78 nudivirus genes (8, 27). Most reside in six clusters on three chromosomes, while 13 are dispersed. Sixteen of the 21 core genes shared with baculoviruses are present, including the following: *helicase*, required for replication of baculovirus genomes (28); *lef-4, lef-5, lef-8, lef-9,* and *p47,* which encode subunits of a DNA-dependent RNA polymerase that transcribes other viral genes (26, 29); and several *pif* genes that encode envelope proteins (30). Two shared core genes that are known to encode capsid proteins in baculoviruses and nudiviruses (*vp39* and *38K*) (5, 26) are pseudogenized in *V. canescens*, which likely explains why VcVLPs lack a capsid (31). Reciprocally, RNAi assays indicate the nudivirus-derived RNA polymerase subunits in *V. canescens* retain conserved functions by producing a holoenzyme that transcribes other DEV genes (32).

Most of the other nudivirus genes in the *V. canescens* genome share no sequence similarity with other genes and are thus named by the OrNV homolog they are most closely related to (8, 27). Some of these genes are alphanudivirus-specific, others are in some but not all sequenced nudiviruses, while five are core genes present in all sequenced nudiviruses but not baculoviruses (8, 27). Three of these genes are single copy (*OrNVorf18-like*, *OrNVorf61-like*, and *OrNVorf76-like*), while *OrNVorf41-like* has expanded into a six-member gene family and *OrNVorf47-like* has expanded into a three-member family (27). The aforementioned proteomic analysis of VcVLPs detected proteins corresponding to *OrNVorf61-like* and some of the *OrNVorf47-like* and *OrNVorf41-like* family members, but did not detect products from *OrNVorf18-like* or *OrNVorf76-like* (8). Here, we conducted studies to further characterize these nudivirus core genes. We report that *OrNVorf18-like,* two *OrNVorf41-*like family members, and *OrNVorf61-like* are required for normal VcVLP formation, while the *OrNVorf47-like* family is essential for VcVLP attachment to eggs. We also show that each of these genes is required for protecting *V. canescens* eggs from encapsulation.

## RESULTS

### Each of the *OrNV-like* nudivirus core genes encodes full-length proteins

Nudivirus, baculovirus, and BV genes with replication functions are transcribed in temporally ordered cascades (4, 15, 26, 33, 34). Early genes expressed at the beginning of a replication cycle include the RNA polymerase subunits, while many late genes are transcribed by the viral RNA polymerase and encode virion components. Transcriptome data similarly classify the RNA polymerase subunits and select other *V. canescens* DEV genes as early because they are upregulated in the ovaries of younger pupae, while the five genes we focused this study on, plus several others, are classified as late because they are upregulated in mid-stage pupae (8, 32). We began this study by asking if any of the nudivirus core gene products we prioritized for investigation had transmembrane domains, as might be expected if they encode VLP structural proteins. A single hydrophobic domain was present in the N-terminus of OrNVorf47-like-3 and OrNVorf76-like, while internal hydrophobic domains were present in OrNVorf41-like-1-6 and OrNVorf61-like (Fig. S1). In contrast, no hydrophobic domains were present in OrNVorf18-like, OrNVorf47-like-1, or OrNVorf47-like-2. None of these domains were predicted to be signal peptides by SignalP 6.0, while all were predicted to form alpha transmembrane domains by DeepTMHMM-1.0. Alignments indicated each of these genes was full length and retained conserved residues when compared to corresponding homologs in OrNV and other nudiviruses (Fig. S2 to S6). The short length of *OrNVorf41-like* family members in *V. canescens* made phylogenetic resolution of relationships difficult (Fig. S2). In contrast, results well-supported clustering of the three *OrNVorf47-like* family members, which suggested this family arose by duplication (Fig. S3). Each of the genes in *V. canescens* also clustered most closely to homologs in alphanudiviruses, which supported earlier conclusions that VcVLPs evolved from a virus in this genus (8).

### VcVLP morphogenesis proceeds through three phases in females injected with ds-*egfp*

**Fig 1:**
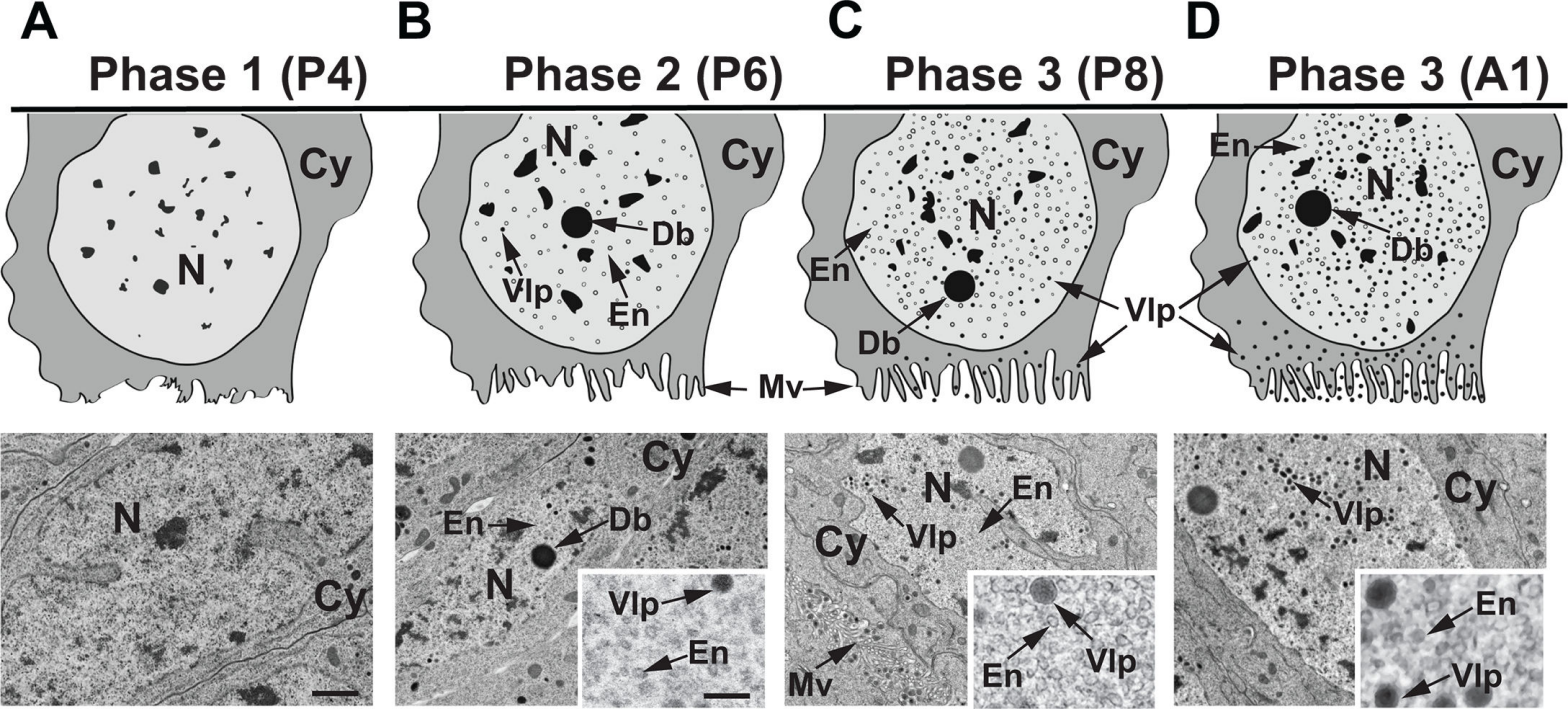
VcVLP morphogenesis in calyx cells from different aged pupae or newly emerged adults that were injected with *ds-egfp* when P1 pupae. The upper panel shows cartoons of calyx cells, while the lower panel shows corresponding TEM images at lower and higher magnification (inserts). (**A**) Phase 1 calyx cell from a 4-day-old pupa (P4) consisting of a nucleus (N) and cytoplasm (Cy) but with no VLP components present. (**B**) Phase 2 calyx cell from a P6 pupa where dense bodies (Db), VLP envelopes (En), and small numbers of mature VLPs containing electron-dense material (VLP) first appear in the nucleus. (**C**) Phase 3 calyx cell from a P8 pupa where an abundance of VLP envelopes and mature VLPs are present throughout the nucleus. Mature VLPs also exit the nucleus, migrate through the cytoplasm, and bud from microvilli (Mv) into the calyx lumen. (**D**) Phase 3 calyx cell from a newly emerged adult (A1) where the abundance of mature VLPs in the nucleus and calyx lumen further increases. Scale bar in low-magnification TEM image = 1 µm with other low-magnification images the same. Scale bar in the higher-magnification TEM image = 100 nm with other higher-magnification images also the same.

Previous TEM data describe VcVLPs in calyx cells from older pupae and newly emerged adults (8, 22, 23, 32). Calyx cell nuclei contain structures originally named dense bodies, VLP envelopes, and mature VLPs that are distinguished by electron-dense material that is packaged into envelopes (22, 23). Mature VLPs exit calyx cell nuclei, migrate through the cytoplasm, and bud through the plasma membrane into the calyx lumen (8, 22, 23). Rearing conditions for *V. canescens* in our laboratory result in an 8 day pupal stage (P1–P8) followed by emergence of day 1 adult females (A1). In preparation for RNAi knockdown experiments, we designed a double-stranded (ds) RNA to *egfp* as a negative control treatment. We first used ds-*egfp* by injecting it into day 1 *V*. *canescens* pupae (P1) to assess whether it had any effects on the duration of the pupal stage and to characterize the chronology of VcVLP morphogenesis, which had not previously been assessed. No differences were observed in the duration of the pupal stage, which remained 8 days in duration. Dissection of ovaries from pupae at different times post-treatment followed by TEM indicated no VLP components were present in calyx cell nuclei from P1–P4 pupae (Fig. 1A). We referred to this period as Phase 1. Phase 2 was distinguished by the appearance of dense bodies, a few VLP envelopes, and a few mature VLPs in calyx cell nuclei. These events occurred in P6 pupae (Fig. 1B). Phase 3 was distinguished by the presence of dense bodies plus large numbers of VLP envelopes and mature VLPs in the nuclei of most calyx cells in P8 pupae and A1 adults (Fig. 1C and D; Fig. S7A). VLP envelopes in the process of accumulating electron-dense material along with empty envelopes and mature VLPs were often observed near dense bodies during Phase 3 (Fig. S7B). However, large numbers of empty envelopes and mature VLPs were also present elsewhere in the nucleus (Fig. S7C). Mature VLPs exited nuclei by budding through the nuclear membrane, which results in acquisition of a second membrane (Fig. S7D). This second membrane is then lost as VLPs migrate through the cytoplasm to microvilli where they egress into the calyx lumen (Fig. S7E). We thus concluded injection of ds-*egfp* does not alter the duration of the pupal stage or the morphology of dense bodies, VLP envelopes, and mature VLPs when compared to previously published descriptions of calyx cells from untreated *V. canescens* females.

### RNAi knockdown of *OrNVorf61-like* causes alterations in VLP morphogenesis, but knockdown of *OrNVorf76-like* does not

**Fig 2:**
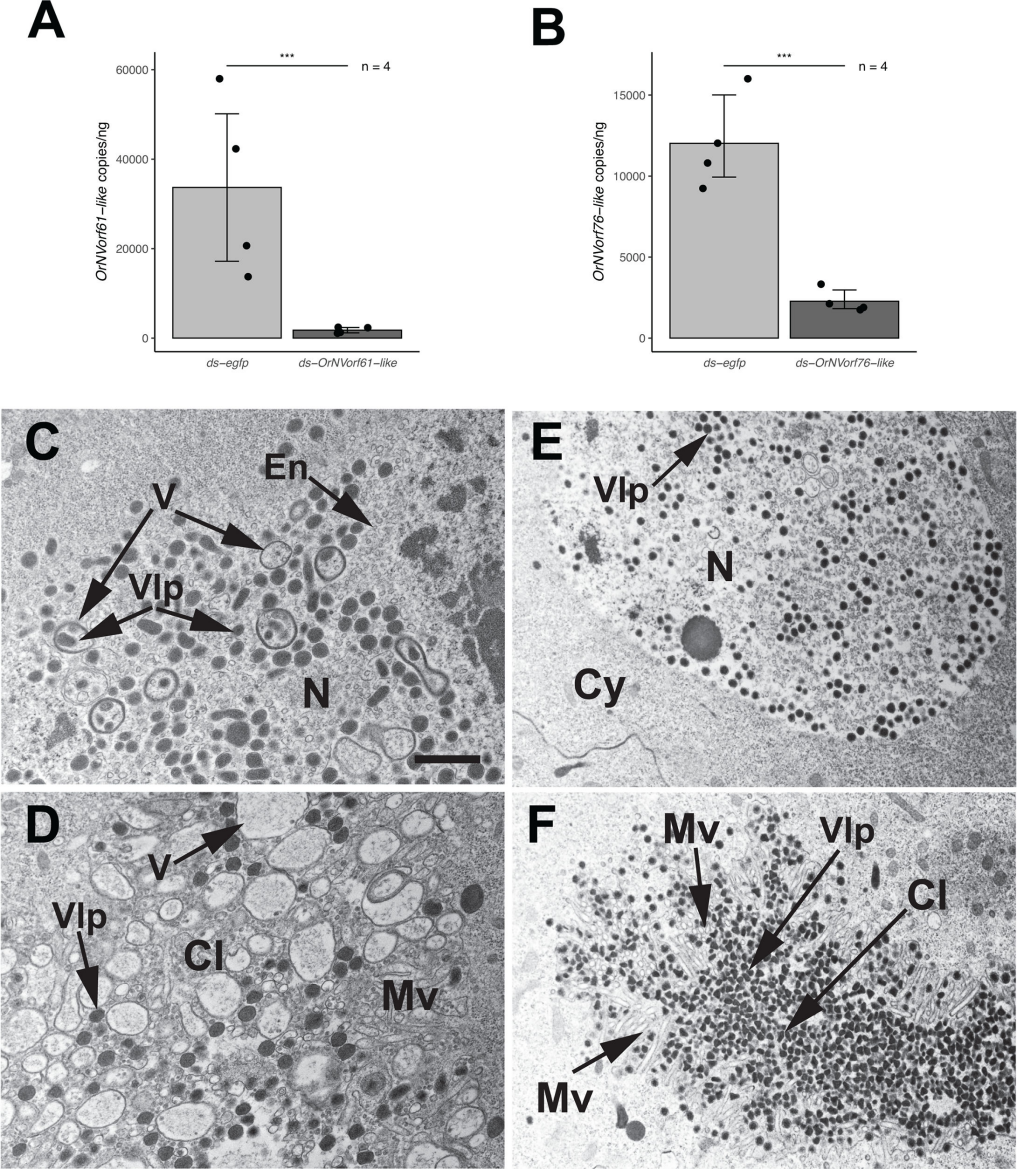
RNAi knockdown of *OrNVorf61-like* and *OrNVorf76-like* and effects on VLP formation. (**A**) Transcript abundance of *OrNVorf61-like* per ng of total RNA in ovaries from A1 females injected with ds-*OrNVorf61-like* or ds-*egfp* (control) as P1 pupae. Bars show mean transcript copy per ng of total RNA ± 1 standard error, solid circles and N values indicate number of females examined, and asterisks indicate treatments significantly differed (two-tailed *t*-test, ***, *P* < 0.001). (**B**) Transcript abundance of *OrNVorf76-like* per ng of total RNA in ovaries from A1 females injected with ds-*OrNVorf76-like* or ds-*egfp* (control) as P1 pupae. Bars, sample sizes, and statistical differences between treatments (*** *P* < 0*.*001) as defined in A. (**C**) TEM image of an ovary calyx cell from an A1 female after *OrNVorf61-like* knockdown showing the presence of circularized VLP envelopes (En) throughout the nucleus (N), while mature VLPs filled with electron dense material are larger than VLPs in control females (see Fig. 1; Fig. S8) and sometimes surrounded by vesicles (V). (**D**) TEM image of the calyx lumen (Cl) from an A1 female after *OrNVorf61-like* knockdown. Microvilli (Mv) from a calyx cell project into the lumen, which contains enlarged VLPs and vesicles. (**E, F**) TEM images of an ovary calyx cell or the calyx lumen from an A1 female after *OrNVorf76-like* knockdown. The nucleus in E contains an abundance of envelopes and mature VLPs with normal morphology, while the calyx lumen in F also contains an abundance of mature VLPs with a normal morphology. Scale bar in C = 600 nm with images in D–F at the same magnification.

Detection of OrNVorf61-like, OrNVorf47-like-3, and two OrNVorf41-like family members (−1 and −2) in VcVLPs by proteomic analysis (8), together with most of these proteins also containing transmembrane domains, suggested each could be a structural component. OrNVorf76-like and the other four OrNVorf41-like family members were not detected in VcVLPs (8), but the presence of transmembrane domains in each suggested they could also have structural roles that are required for normal VLP formation. We tested this by designing dsRNAs and focusing first on the two single-copy genes (*OrNVorf61-like* and *OrNVorf76-like*). Each was injected into P1 females followed by collecting the paired ovaries from A1 adults. One ovary per individual was used in qRT-PCR assays, which showed that each dsRNA significantly reduced transcript abundance of its target gene (80%–95%) when compared to control P1 females that were injected with ds-*egfp* (Fig. 2A and B). We then used TEM to examine calyx cell nuclei and the calyx lumen. Knockdown of *OrNVorf61-like* resulted in calyx cell nuclei that contained VLP envelopes like calyx cells from control females treated with ds-*egfp* (Fig. 2C). However, mature VLPs in calyx cell nuclei were larger, less spherical in shape, and less abundant than in control females, while numerous vesicles that sometimes enveloped mature VLPs were also present (Fig. 2C). In contrast, no vesicles in calyx cell nuclei were observed to traverse the nuclear membrane into the cytoplasm. Examination of the calyx cell lumen showed that some enlarged, mature VLPs were present, but the abundance was consistently lower than the number of mature VLPs that were present in the calyx lumen of control females (Fig. 2D). Numerous vesicles and other cellular debris were also present. However, none of these vesicles surrounded mature VLPs. Unlike the preceding outcome, knockdown of *OrNVorf76-like* resulted in no visible alterations in calyx cells or the calyx lumen when compared to control females injected with ds-*egfp* (Fig. 2E and F). Like control females, the high density of VLPs in the calyx lumen after *OrNVorf76-like* knockdown females was also associated with little or no visible cellular debris (Fig. 2F).

### RNAi knockdown of *OrNVorf41-like-1* and −*2* also alters VLP morphogenesis

**Fig 3:**
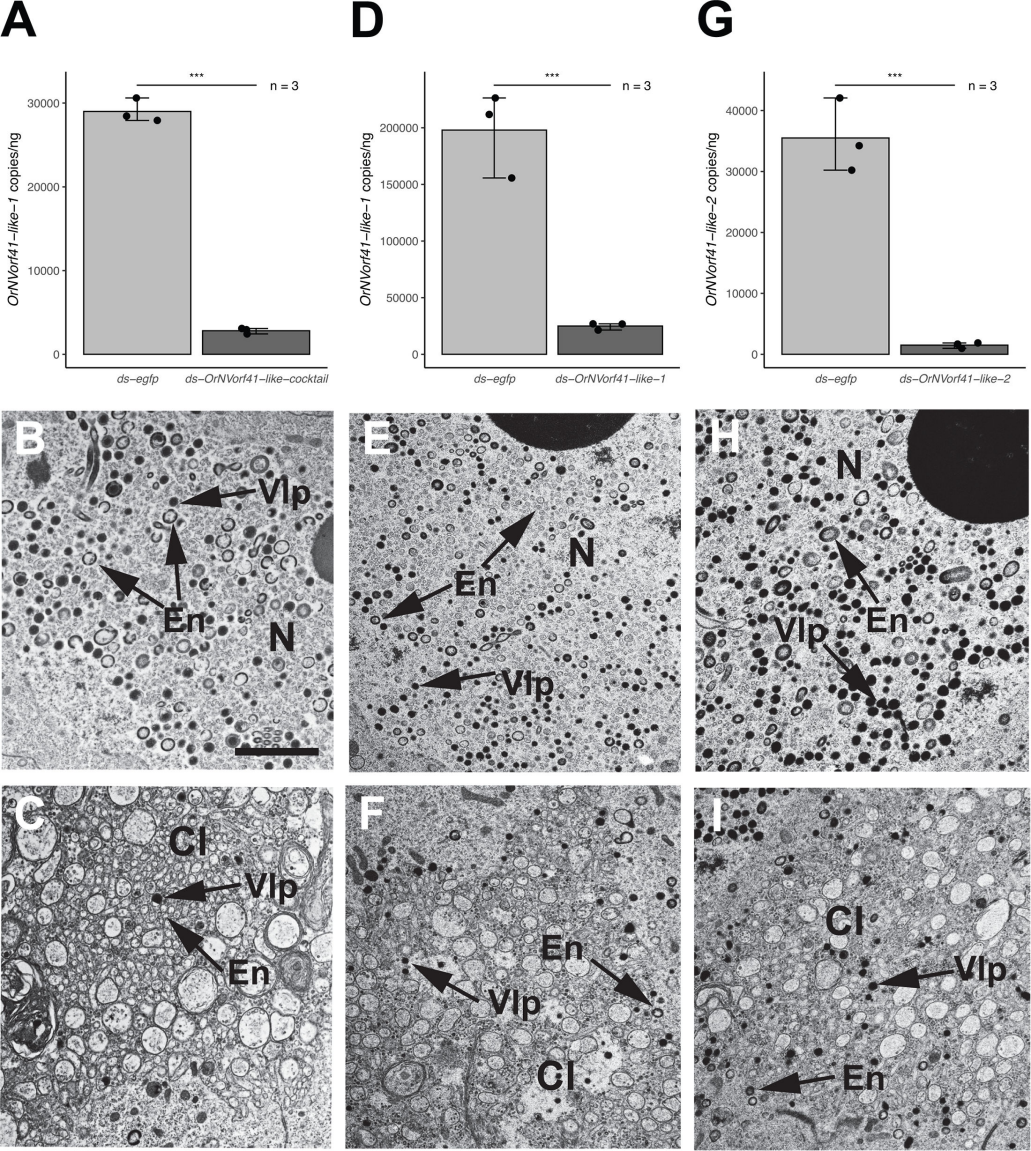
RNAi knockdown of the *OrNVorf41-like* family, *OrNVorf41-like-1,* or *OrNVorf41-like-2* and effects on VLP formation. (**A**) Transcript abundance of *OrNVorf41-like-1* per ng of total RNA in ovaries from A1 females injected with a ds-*OrNVorf41-like-*cocktail or ds-*egfp* as P1 pupae. Bars, error values, sample sizes, and asterisks as defined in Fig. 2 (two-tailed *t*-test, *** *P* < 0.001). (**B**) TEM image of an ovary calyx cell nucleus (N) from an A1 female after treatment with ds-*OrNVorf41-like-*cocktail. Many VLP envelopes (En) are enlarged and thickened, while mature VLPs are less abundant than in control females (see Fig. 1; Fig. S8) and also sometimes surrounded by vesicles (V). (**C**) TEM image of the calyx lumen (Cl) from an A1 female after treatment with ds-*OrNVorf41-like-*cocktail. Large numbers of empty VLP envelopes are present, but few mature VLPs are observed. (**D**) Transcript abundance of *OrNVorf41-like-1* per ng of total RNA in ovaries from A1 females injected with ds-*OrNVorf41-like-1* or ds-*egfp* as P1 pupae. Bars, error values, sample sizes, and asterisks as defined in A (*** *P* < 0.001). (**E, F**) TEM images of an ovary calyx cell nucleus or the calyx lumen after treatment with ds-*OrNVorf41-like-1*. (**G**) Transcript abundance of *OrNVorf41-like-2* per ng of total RNA in ovaries from A1 females injected with ds-*OrNVorf41-like-2* or ds-*egfp* as P1 pupae. Bars, error values, sample sizes, and asterisks as defined in A (*** *P* < 0.001). (**H, I**) TEM images of an ovary calyx cell nucleus or the calyx lumen after treatment with ds-*OrNVorf41-like-2*. Scale bar in B = 600 nm with other TEM images at the same magnification.

We next designed a cocktail of dsRNAs that were co-injected into P1 pupae to knock down all *OrNVorf41-like* family members. We assessed the efficacy of this cocktail by measuring the transcript abundance of one family member (*OrNVorf41-like-1*), which was reduced more than 85% in A1 females when compared to the negative control (Fig. 3A). Examination of calyx cell nuclei from A1 females showed that some empty VLP envelopes and mature VLPs with a normal morphology were present (Fig. 3B). However, many of the VLP envelopes in calyx cell nuclei were also larger and more electron-dense than the envelopes in control females (Fig. 3B). The calyx lumen from females treated with the ds-*OrNVorf41-like* cocktail contained few mature VLPs, while an abundance of vesicles and other cellular debris was visible (Fig. 3C). We next injected dsRNAs specific for each *OrNVorf41-like* family member into P1 pupae, followed by assessment of A1 adults. Knockdown of *OrNVorf41-like-1* or *−2* resulted in the same alterations observed in females treated with ds-*OrNVorf41-like*-cocktail, with the exception that a few more mature VLPs were consistently observed in the calyx lumen versus females injected with the ds*-OrNVorf41-like-*cocktail (Fig. 3D through I). In contrast, knockdown of *OrNVorf41-like-3, -4, −5,* or *−6* resulted in no visible alterations in VLP assembly in calyx cell nuclei or the morphology of mature VLPs in nuclei or the calyx lumen (Fig. S8). The dsRNAs designed to knock down *OrNVorf41-like-1* or *−2* were made to regions of these paralogs that share no homology. However, because phenotypes after treatment were very similar, we checked for off-target effects by comparing transcript abundances in A1 females treated with ds-*OrNVorf41-like-1* or *−2* to females treated with ds-*egfp*. Results indicated no off-target effects occurred since ds*-OrNVorf41-like-1* significantly reduced the transcript abundance of *OrNVorf41-like-1,* but not *OrNVorf41-like-2,* while ds*-OrNVorf41-like-2* significantly reduced *OrNVorf41-like-2,* but not *OrNVorf41-like-1* (Fig. S9). We thus concluded *OrNVorf41-like-1* and *−2* are both required for normal VLP formation, whereas *OrNVorf41-like-3 to -6* are not.

### RNAi knockdown of *OrNVorf47-like* family members has no effect on VLP formation, whereas knockdown of *OrNVorf18-like* inhibits VLP formation

**Fig 4:**
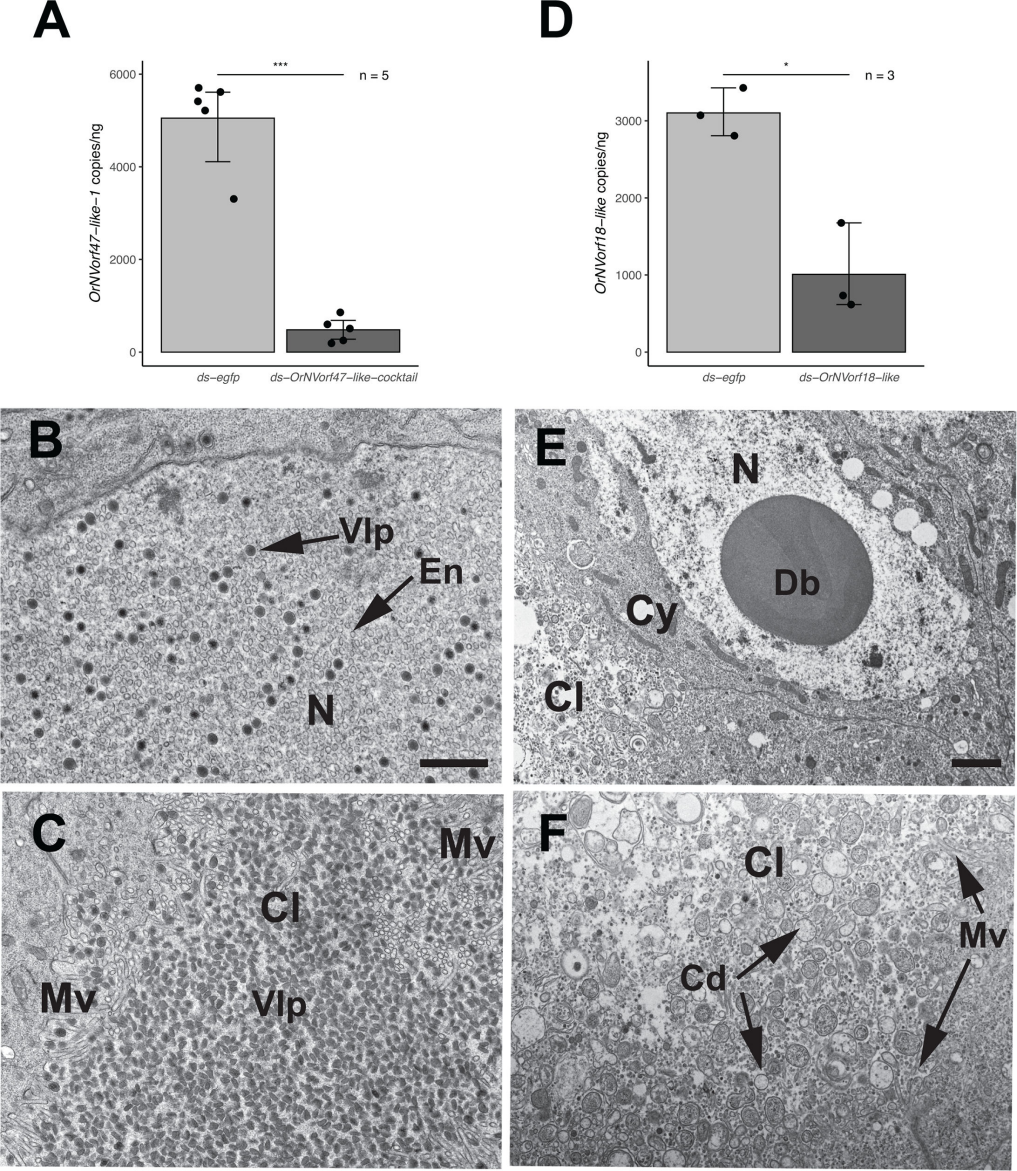
RNAi knockdown of the *OrNVorf47-like* family and *OrNVorf18-like* and effects on VLP formation. (**A**) Transcript abundance of *OrNVorf47-like-1* per ng of total RNA in ovaries from A1 females injected with ds-*egfp* or ds-*OrNVorf47-like-*cocktail as P1 pupae. Bars, error values, sample sizes, and asterisks, as defined in Fig. 2 (two-tailed *t*-test, *** *P* < 0.001). (**B**) TEM image of an ovary calyx cell nucleus (N) from an A1 female after treatment with ds-*OrNVorf47-like-*cocktail. VLP envelopes (En) and mature VLPs (VLP) show no alterations when compared to control females (see Fig. 1; Fig. S8). (**C**) TEM image of the calyx lumen (Cl) from an A1 female after treatment with ds-*OrNVorf47-like-*cocktail. Microvilli (Mv) projecting from neighboring calyx cells are visible along with an abundance of morphologically normal VLPs. (**D**) Transcript abundance of *OrNVorf18-like* per ng of total RNA in ovaries from A1 females injected with ds-*egfp* or ds-*OrNVorf18-like* as P1 pupae. Bars, error values, sample sizes, and asterisks as defined in A (* *P* < 0.05). (**E, F**) TEM images of an ovary calyx cell nucleus or the calyx lumen after treatment with ds-*OrNVorf18-like*. The nucleus in E contains an enlarged dense body (Db), but no VLP envelopes or mature VLPs, while the calyx lumen in F contains cellular debris (Cd) but also lacks VLPs. Scale bar in B = 600 nm with TEM image in C and F at the same magnification. Scale bar in E = 600 nm.

The last genes we examined were the three-member *OrNVorf47-like* family, where one paralog has a transmembrane domain (OrNVorf47-like-3) but two do not (OrNVorf47-like-1 and −2), and *OrNVorf18-like,* which also lacks a transmembrane domain. As with the *OrNVorf41-like* family, we first injected a ds-*OrNVorf47-like* cocktail into P1 pupae followed by assessment of knockdown of one family member (*OrNVorf41-like-1*) in the ovaries of A1 adults. Transcript abundance of this family member was reduced more than 85% when compared to females treated with ds-*egfp,* but TEM data showed no alterations in VLP formation in calyx cell nuclei or the accumulation of mature VLPs in the calyx lumen (Fig. 4A through C). In contrast, knockdown of ds-*OrNVorf18-like* resulted in most calyx cell nuclei containing no VLP envelopes or mature VLPs, while a single dense body that was much larger than the dense bodies in calyx cell nuclei from untreated females or females treated with ds-*egfp* was present (Fig. 4E). In turn, the calyx lumen contained an abundance of vesicles and other debris but almost no VLPs (Fig. 4F). Examination of calyx cells showed extensive budding from the plasma membrane, which formed vesicles of varying size that were either empty or contained cytoplasm (Fig. S10). The vesicles and other cellular debris in the calyx lumen were also similar to the debris observed in the calyx lumen after knockdown of *OrNVorf61-like* and *OrNVorf41-like-1* and *−2* (see Fig. 2D, 3C, F and I).

### Knockdown of *OrNVorf47-like* gene family and several other nudivirus core genes disables VLP attachment to *V. canescens* eggs

**Fig 5:**
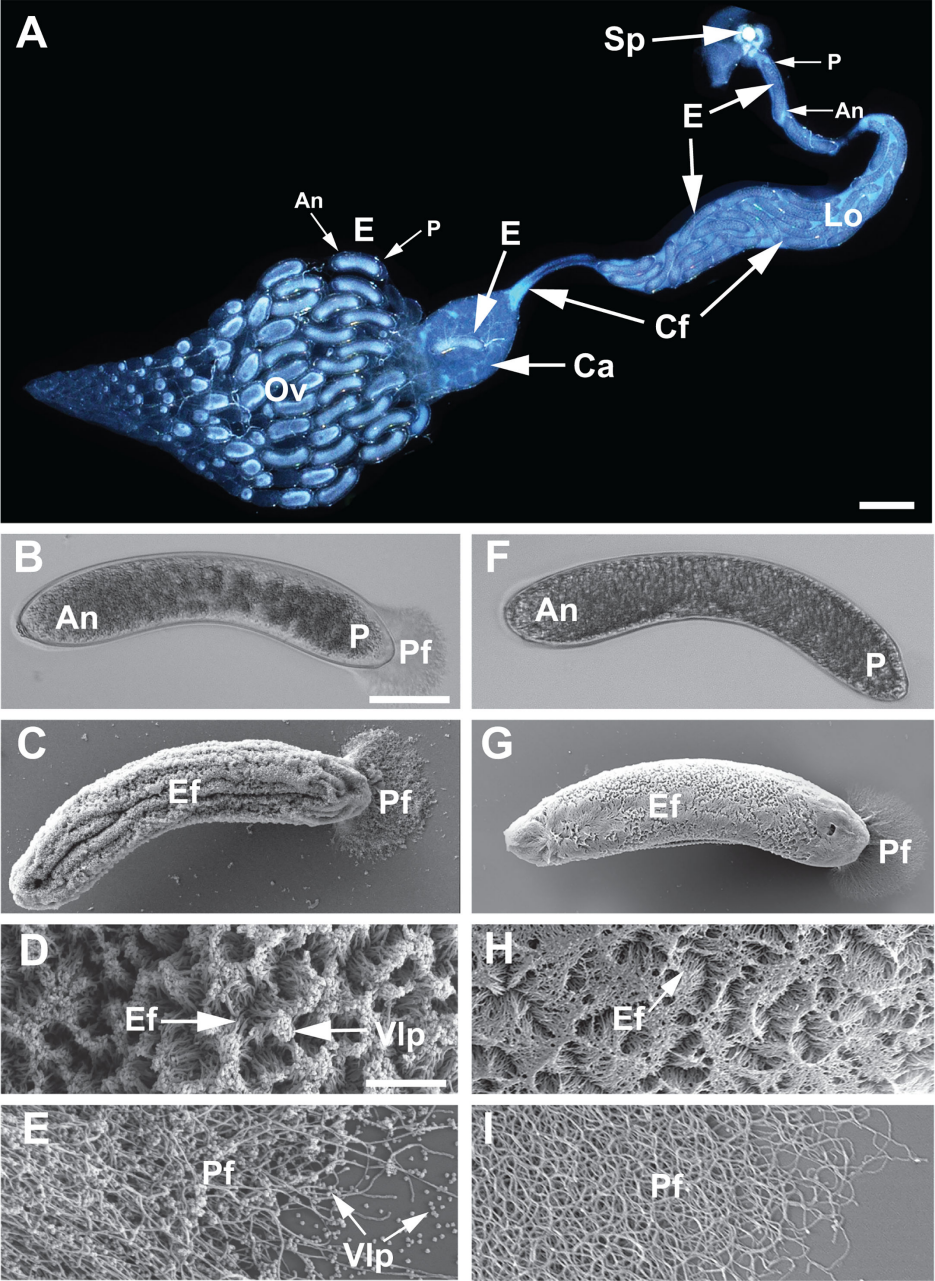
*V. canescens* eggs from A1 females injected with ds-*egfp* or ds-*OrNVorf47-like-*cocktail as P1 pupae. (**A**) Low-magnification light microscopy image showing an ovary (Ov), calyx (Ca), lateral oviduct (Lo), and spermatheca (Sp). The other ovary and associated structures were removed before photographing the image. The distal end of the ovary is oriented to the left, while the proximal end of the ovary where ovarioles containing mature eggs (E) connect to the calyx is to the right. All eggs in the reproductive tract are oriented with anterior (An) to the left and posterior (P) to the right. One egg is in the calyx lumen, while numerous eggs are in the lateral oviduct. Blue-colored calyx fluid (Cf) containing mature VLPs also exits the calyx lumen and surrounds eggs in the lateral oviduct. Image courtesy of Logan Moore, reproduced with permission. (**B**) Light microscopy and (**C**) SEM image of eggs collected from the lateral oviducts of females treated with ds-*egfp.* The anterior of each egg is oriented to the left. Egg filaments (Ef) visible by SEM extend from the surface of the egg, while the fan-shaped structure visible by light microscopy at the posterior end of eggs is formed by posterior filaments (Pf). (**D**) Higher-magnification SEM image showing egg filaments. An abundance of mature VLPs (VLP) bind to the tips of egg filaments. (**E**) Higher-magnification SEM image showing VLPs also bind to posterior filaments. (**F**) Light microscopy and (**G**) SEM image of eggs from females treated with ds-*OrNVorf47-like-*cocktail*.* Eggs are oriented as in B and C, with posterior filaments visible by SEM. (**H, I**) Higher-magnification SEM images showing egg and posterior filaments with almost no VLPs attached. Scale bar in A = 220 µm. Scale bar in B = 20 µm with images in C, F, and G at the same magnification. Scale bar in D = 5 µm with images in E, H, and I at the same magnification. Note that debris from the background of the ovary image shown in (**A**) was removed using Photoshop; the original image is presented as Fig. S16.

Follicle cells surrounding oocytes secrete a chorion upon reaching the proximal ovarioles, followed by deposition of a material that forms projections on the surface of the chorion when viewed by TEM (22, 35). These projections were also previously noted to extend from the posterior end of eggs to form a fan-like structure when viewed by light microscopy (22). Follicle cells then degenerate, which is followed by eggs passing through the calyx lumen and storage in the lateral oviducts (22, 24). TEM detected mature VLPs in proximity to eggs in the calyx lumen and lateral oviducts but could not determine if VLPs bind to the surface of eggs (22). To address this question, we first used low-magnification light microscopy to image the reproductive tract from A1 females injected with ds-*egfp.* Ovaries contained an abundance of eggs in different stages of development, with mature eggs individually passing through the calyx before storage in the lateral oviducts (Fig. 5A). When viewed by light microscopy, mature VLPs appear as blue-colored “calyx fluid,” which was observed to exit the calyx lumen and surround stored eggs in the lateral oviducts (Fig. 5A). The fan-shaped structure extending from the posterior of eggs was also visible by light microscopy, whereas no other material on the surface of eggs could be seen (Fig. 5B). In contrast, scanning electron microscopy (SEM) clearly showed that material forming 3–5-µm-long filaments covered the surface of eggs, while the fan-like structure visible by light microscopy was formed by the same material forming 20–40-µm-long filaments (Fig. 5C through E). The distinct differences in the length led us to name the former egg filaments and the latter posterior filaments. SEM also clearly showed that large numbers of VLPs were bound to the distal tips of egg filaments and along the length of posterior filaments (Fig. 5D and E).

We next examined eggs from females injected at P1 with the ds-*OrNVorf47-like*-cocktail, which caused no alterations in the formation or accumulation of mature VLPs in the calyx lumen. Stored eggs from the lateral oviducts looked normal when examined by light microscopy, with the exception that no fan-shaped structure was visible (Fig. 5F). SEM showed that morphologically normal egg and posterior filaments were present, but almost no VLPs were attached (Fig. 5F through I). This finding strongly suggested an essential role for *OrNVorf47-like* gene products in binding of VLPs to filaments, while also suggesting bound VLPs are required to see the fan-like structure that is visible by phase-contrast microscopy. Alignments identified no definitive options for designing dsRNAs that did not result in 20 bp domains that overlapped with domains present in other family members. We thus expected each dsRNA we designed would cross-silence other family members, which occurred when we conducted an off-target assay (Fig. S11). Our results, therefore, identify the *OrNVorf47-like* family as essential for VLP binding to egg and posterior filaments but do not distinguish whether all or only certain paralogs are required.

We also assessed whether bound VLPs are required to see the posterior fan-like structure on eggs when viewed by light microscopy by using females injected at P1 with ds-*OrNVorf61-like,* ds-*OrNVorf41-like-*cocktail, or ds-*OrNVorf18-like.* Since the knockdown of each of these genes caused defects that reduced or eliminated VLPs from the calyx lumen, we expected each to also result in few or no VLPs bound to filaments on eggs. This was fully supported by light microscopy and SEM analysis of eggs (Fig. S12A through F). In contrast, eggs from females treated with ds-*OrfNVorf76-like*, which caused no alterations in VLP morphogenesis or accumulation in the calyx lumen, were indistinguishable from control eggs (Fig. S12G and H). Treating females with ds-RNAs specific for each *OrNVorf41-like* family member followed by inspection of eggs from A1 adults similarly showed that knockdown of *OrNVorf41-like-1* or −2 resulted in loss of visibility of the fan-shaped structure, whereas knockdown of *OrNVorf41-like-3, -4, −5,* or *−6* did not (Fig. S13A through F). This outcome was consistent with *OrNVorf41-like-1* or −2 also being the only family members that adversely affected VLP formation and accumulation in the calyx lumen. Thus, the *OrNVorf47-like* family plus all of the other nudivirus core genes that adversely affected VLP formation resulted in few or no VLPs binding to eggs. We further concluded posterior filaments were only visible by phase-contrast microscopy if large numbers of VLPs were attached.

### Host hemocytes encapsulate most eggs lacking bound VLPs

**Fig 6:**
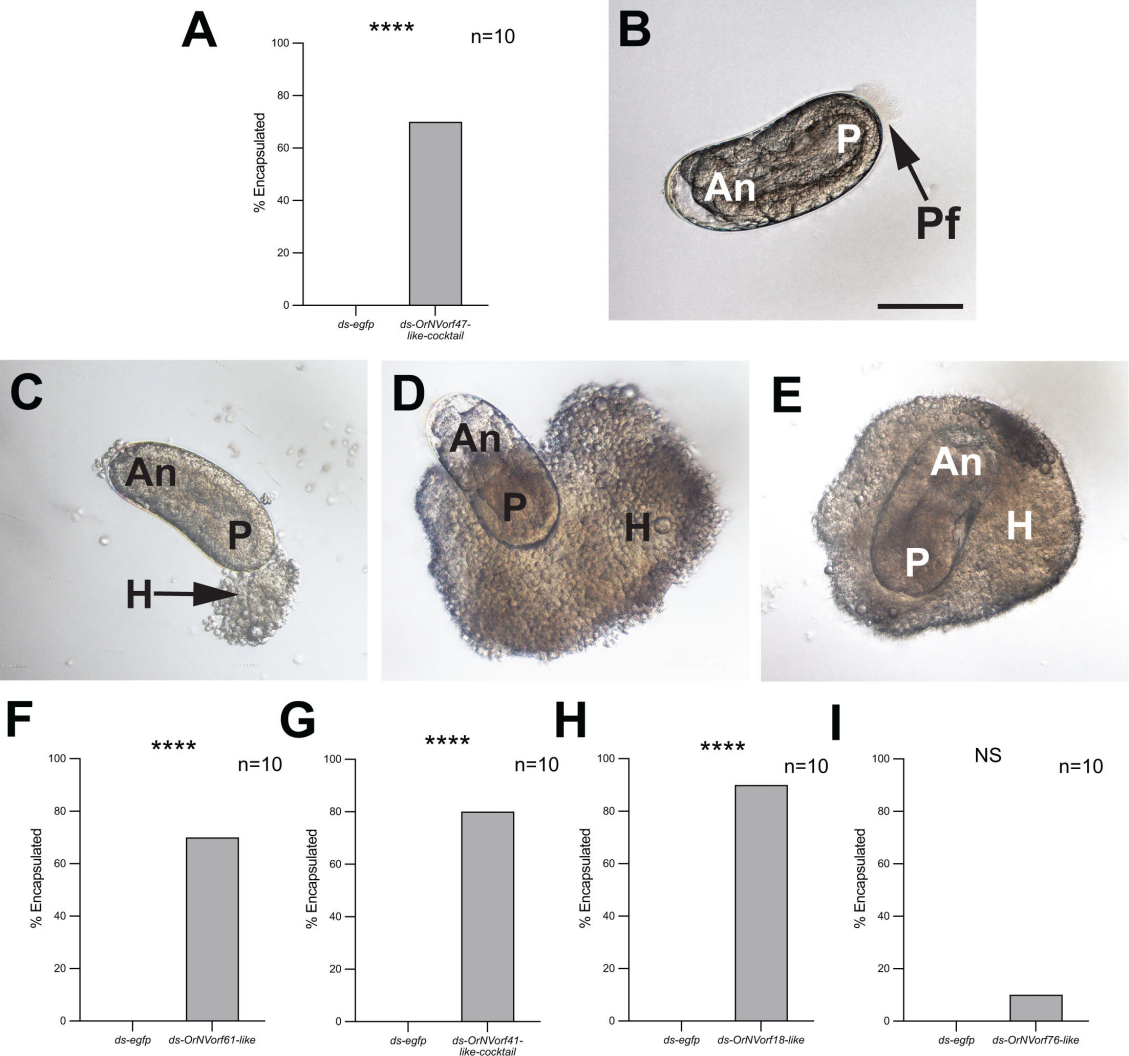
*E. kuehniella* hemocytes encapsulate eggs without VLPs attached to filaments. (**A**) Percentage of hosts with a partially or fully encapsulated egg 48 h post-parasitism by a female treated with ds-*egfp* or ds-*OrNVorf47-like-*cocktail. Bars show percent encapsulation, n values indicate number of hosts for each treatment, and asterisks indicate significance (Fisher’s exact test, **** *P* < 0.0001). (**B**) Light micrograph of a 48 h post-parasitism egg laid by a ds-*egfp* treated female. Anterior (An) is to the left and posterior (P) is to the right. The posterior filament (Pf) is also visible. (**C**) A 48 h post-parasitism egg laid by a ds*-OrNVorf47-like-*cocktail-treated female in the early stage of being encapsulated. Hemocytes (H) are primarily attached to the posterior end of the egg. (**D**) A 48 h post-parasitism egg laid by a ds-*OrNVorf47-like-*cocktail-treated female, where large numbers of hemocytes surround the posterior half of the egg. (**E**) A 48 h post-parasitism egg laid by a ds-*OrNVorf47-like-*cocktail-treated female that is fully encapsulated. Scale bar in A = 100 µm with the images in C–E at the same magnification. (**F–I**) Percentage of hosts with a partially or fully encapsulated egg 48 h post-parasitism by a female treated with ds-*egfp* or ds-*OrNVorf61-like,* ds-*OrNVorf41-like-*cocktail, and ds-*OrNVorf18-like* or ds-*OrNVorf76-like*. Bars, sample sizes, and asterisks as defined in A (**** *P* < 0.0001, NS nonsignificant).

A key host defense response against parasitoid wasps is encapsulation: a process whereby immune cells (hemocytes) surround and kill eggs or larvae (36, 37). Previous experiments showed that *E. kuehniella* hemocytes do not encapsulate eggs that have passed through the calyx lumen and are stored in the lateral oviducts but do encapsulate eggs collected from ovarioles before passage through the calyx lumen (23, 24, 38). This outcome formed the basis for concluding VLPs protect eggs from encapsulation. In contrast, results from knocking down the *OrNVorf47-like* family indicate VLPs cannot bind to eggs but otherwise show no alterations in morphology or abundance. We thus asked if eggs are only protected from encapsulation if VLPs are bound to filaments by injecting females with ds-*egfp* or ds-*OrNVorf47-like* cocktail as P1 pupae followed by allowing A1 adults to parasitize fifth-instar *E. kuehniella* larvae. Dissection of hosts 48 h later showed that no hosts parasitized by control females contained encapsulated eggs, whereas 70% of hosts parasitized by treatment females contained partially or fully encapsulated eggs (Fig. 6A). Phase-contrast microscopy showed that no hemocytes were present on eggs laid by control females while posterior filaments were also still visible, which strongly suggested VLPs remained present (Fig. 6B). In contrast, eggs laid by treatment wasps, which lack visible posterior filaments, showed a progression of encapsulation states at 48 h. Some eggs lacked any bound hemocytes, while others showed hemocytes preferentially bound to the posterior end of the egg or that extended from the posterior to the anterior end (Fig. 6C and D). Some eggs were also fully surrounded by hemocytes that formed a complete capsule (Fig. 6E). Given these results, we also compared encapsulation of eggs laid by females treated with ds-*OrNVorf61-like,* ds-*OrNVorf41-like-*cocktail*,* or ds-*OrNVorf18-like,* which each disabled VLP production in calyx cells and binding to eggs. Again, almost no eggs laid by control females treated with ds-*egfp* were encapsulated, while most eggs laid by treatment females were encapsulated (Fig. 6F through H). In contrast, females treated with ds-*OrNVorf76-like,* which did not affect VLP production or binding to filaments, laid eggs like control females that were almost never encapsulated (Fig. 6I).

## DISCUSSION

The alphanudivirus-derived DEV in *V. canescens* produces VcVLPs, which associate with eggs that are resistant to encapsulation in permissive hosts like *E. kuehniella* (8, 22–24, 38). Our goal in conducting this study was to identify genes unique to nudiviruses that are required for VLP assembly, egg association, and/or protection from host hemocytes. Nudivirus genes not shared with baculoviruses lack any sequence homology to suggest what their functions might be. We thus focused this study on the five genes in the *V. canescens* genome that all nudiviruses encode but are not core genes that are shared with baculoviruses. Our premise for selecting these targets was that each is likely essential to the VcVLP ancestor and has been maintained in *V. canescens* because they remain essential for VLP production and/or function.

Proteomic analysis of nudivirus virions is currently restricted to one species (*Tipula oleracea* nudivirus [ToNV]), where homologs of OrNVorf41, OrNVorf47, OrNVorf61, and OrNVorf76 are detected (39). This finding suggests each could be a required structural protein. Detection of OrNVorf61-like, OrNVorf41-like-1 and −2, and OrNVorf47-like-1 and −3 in VcVLPs (8) along with the presence of transmembrane domains in most of these proteins suggests each could also retain structural functions as VLP envelope components. In contrast, diversification of OrNVorf41-like and OrNVorf47-like into multimember families in *V. canescens* could suggest some paralogs retain ancestral functions while others have been repurposed or degraded. Detection of an OrNVorf76 homolog in ToNV virions but not VcVLPs could also reflect structural differences between each, while the absence of a transmembrane domain in OrNVorf18-like along with not detecting this protein in VcVLPs or ToNV virions at minimum suggests it is not a structural factor.

We characterized the timing of VcVLP formation in females treated with ds-*egfp* because prior studies primarily focused on morphogenetic events in adult females. Our analysis identified an early stage (Phase 1) during the pupal stage when no VLP components are present in calyx cells, a mid-stage (Phase 2) when VLP components first appear, and a late stage (Phase 3) when large numbers of mature VLPs are present. The timeline for these phases is consistent with a previous transcriptome analysis showing that the RNA polymerase subunits and select other DEV genes are expressed in early-stage pupae while all other DEV genes are upregulated in mid- and late-stage pupae, including several encoding products detected in VLPs (32). Our results indicate that structures originally named dense bodies (23) first appear in calyx cell nuclei during Phase 2 when VLP envelopes also first appear. The morphology of dense bodies somewhat resembles electron-dense virogenic stroma that forms in baculovirus and nudivirus-infected cell nuclei and is known to contain components required for virion assembly and DNA replication (40–42). The close association of dense bodies with VLPs in different stages of maturation during Phase 3 suggests this structure contains factors that are packaged into VLP envelopes: a conclusion supported by immunogold labeling studies that detected some VLP components in dense bodies (8). In contrast, the first VLP envelopes that form during Phase 2 and most envelopes present in Phase 3 are not near dense bodies, which suggests dense bodies are not the source of the nudivirus gene products that are envelope components.

We organized our RNAi assays to first assess whether each gene we targeted for study is required to produce VcVLPs with a normal morphology. Our TEM results show that knockdown of *OrNVorf61-like,* two *OrNVorf41-like* family members, and *OrNVorf18-like* results in different defects, while knockdown of *OrNVorf76-like* and the *OrNVorf47-like* family does not*.* The larger but fewer VLPs that form in *OrNVorf61-like* knockdown wasps are consistently associated with formation of vesicles in the nuclei of calyx cells that are never observed in control females. The presence of mature VLPs in these vesicles shares similarities with vesicles that form during morphogenesis of *Oryctes rhinoceros* nudivirus (OrNV) virions (41). However, the vesicles that surround OrNV virions mediate nucleocytoplasmic transport and egress, whereas the vesicles that form after *OrNVorf61-like* knockdown were never observed to exit calyx cell nuclei.

Our assays using a ds-*OrNVorf41-like* cocktail suggest one or more members of this gene family cause defects in VLP envelope size and thickness in calyx cell nuclei plus an increase in the abundance of empty envelopes but a decrease in the abundance of mature VLPs in the calyx lumen. Treatment with ds-*OrNVorf41-like-1* or *−2* alone generates similar defects, which indicates a requirement for both to produce VLPs with a normal morphology rather than full functional redundancy and raises the possibility that these homologous proteins cooperate to properly function. Consistent with causing envelope defects, *OrNVorf41-like* genes in nudiviruses and *V. canescens* share some sequence features with the *11k* gene family present in several large DNA viruses including baculoviruses where family members named Ac145 and Ac150 localize to occlusion-derived virus envelopes (5, 26, 43). The significance of two or more *11k-like* family members in viral genomes or DEVs is unclear, with information from baculoviruses indicating that functional redundancy or compensation of individual gene products can differ depending upon the identity of the infected host (44). Our evidence that *OrNVorf41-like-3, -4, −5,* or *−6* are similarly transcribed as late genes in *V. canescens* calyx cells like *OrNVorf41-like-1* and *−2* (27, 32) but cause no defects when individually knocked down suggests repurposing for still unknown functions or neutral evolution. Knockdown of *OrNVorf18-like* resulted in the strongest defects in VLP morphogenesis with no formation of envelopes or mature VLPs. These alterations strongly suggest an essential role for the protein encoded by *OrNVorf18-like* in envelope formation, although no detection of this gene product in VLPs (8) also suggests it is not a structural protein. We further speculate dense body enlargement associated with *OrNVorf18-like* knockdown reflects additional accumulation of electron-dense products that are normally packaged into envelopes to produce mature VLPs. As earlier noted, knockdown of *OrNVorf18-like* was also associated with an abundance of cellular debris in the lumen that our results indicate is due in part to budding of calyx cell plasma membranes. Cellular debris from dead follicle cells was earlier noted to accumulate in the calyx lumen of *V. canescens* (22, 23). Cellular debris was also readily apparent in the calyx lumen of females after knockdown of *OrNVorf61-like* and *OrNVorf41-like-1* and *−2* where VLP formation was greatly reduced but not fully eliminated. In contrast, no accumulation of cellular debris was observed when we knocked down *OrNVorf76-like* or *OrNVorf41-like-3, -4, -5, and -6,* which caused no visible defects in VLP formation or morphology. These observations collectively suggest blebbing of calyx cell plasma membranes in A1 adults is related to reduced or absent VLP formation, but our results do not identify why this occurs.

We followed our analysis of VLP assembly defects by using SEM to compare the surface of eggs from control and knockdown females for the above treatments. Results advance early TEM observations by showing that VLPs from control females directly bind to the distal tips of egg filaments and along the entire length of posterior filaments. The most significant finding is the discovery that the *OrNVorf47-like* family is not required for normal VLP formation, but fully disables binding of mature VLPs to eggs, which also results in most eggs being encapsulated in *E. kuehniella*. These outcomes are interesting because they indicate the *OrNVorf47-like* family encodes products that VLPs require for binding to egg filaments, while also providing experimental evidence that inhibition of VLP binding disables protection from the host immune system given unbound VLPs in these females are injected into hosts. However, it is also possible the *OrNVorf47-like* family has functions in addition to binding to filaments that prevents host hemocytes from encapsulating eggs. Histochemical assays indicate glucosaminoglycans (GAGs) are present on the filaments coating *V. canescens* eggs (22), while the literature at large indicates a wide variety of proteins bind GAGs (45). Further studies will be needed to determine if *OrNVorf47-like* family members encode GAG-binding proteins and the mechanism(s) underlying why VLP binding to filaments prevents host hemocytes from forming capsules. Shared homology prevented us from being able to knock down individual family members. Thus, future studies will also be needed to determine if all or only certain members of the *OrNVorf47-like* family are required for binding to eggs and immune protection. That knockdown of *OrNVorf61-like, OrNV41orf-like-1* and *−2,* and *OrNVorf18-like* also results in an absence of VLPs on eggs and encapsulation, which is consistent with defects in VLP formation that reduce or prevent VLPs from accumulating in the calyx lumen. In turn, encapsulation of most eggs laid by females where these genes were knocked down is consistent with VLPs being required for protection of eggs from host hemocytes.

In conclusion, reported results provide new insights into the functional roles of four nudivirus core genes in VcVLP formation and function. Our results also likely provide insights into the roles of these genes in nudiviruses, where we hypothesize *OrNVorf18, OrNVorf41,* and *OrNVorf61* are required for envelope formation and *OrNVorf47* may be involved in binding to cells during infection. In contrast, our results do not provide any insights into the function of *OrNVorf76-like,* where knockdown causes no defects in VLP formation, binding to eggs, or protection against encapsulation. Lastly, we note that only some of the nudivirus core genes studied here have been retained by BVs and other DEVs of nudivirus origin that have been identified in parasitoids (5). Which of these genes have been retained versus lost in these other DEV lineages may reflect morphological and functional differences in the virions or VLPs each produces.

## MATERIALS AND METHODS

### Insects

Two permissive hosts for *V. canescens, E. kuehniella* and *Plodia interpunctella* were reared in the laboratory (27). Both species were maintained at 26°C, 40%–62% humidity, and a 12 h light: 12 h dark photoperiod. Larvae were fed a diet consisting of a 2:2:1 ratio (by weight) of chick starter mash, cornmeal, and glycerol. Adults were transferred to jars with screen lids. After mating, females laid eggs in the jars, which were transferred to containers with rearing diet where larvae developed and pupated. *P. interpunctella* was primarily used for maintenance of the *V. canescens* culture, while *E. kuehniella* larvae were used in encapsulation assays because prior studies used this species (22–24). Parasitized larvae for rearing were allowed to pupate. The pupae were then sifted, and the wasps eclosed in a square mesh cage where they were fed honey and water in 0.5% agar.

### Sequence analyses

Homologs of each gene encoded by nudiviruses were identified using Blast-P. Signal peptide and transmembrane domains were identified using SignalP 6.0 and DeepTMHMM-1.0 (https://services.healthtech.dtu.dk/services/DeepTMHMM-1.0/) with default settings (46, 47). Amino acid sequences were aligned with MAFFT using the l-INS-i model (48). Poorly aligned positions were excluded by trimAI V1.3 (strict setting) (49). Substitution models for each gene were determined with ModelTest-NG (50). Maximum likelihood trees were constructed with RAxML-HPC2 via the CIPRES Science Gateway portal (51, 52).

### RNAi assays and quantification of target gene transcript abundances

RNAi assays were performed as previously detailed (14, 15). Briefly, forward and reverse primers with added T7 promoter adapters were designed (Table S1) to generate dsRNAs using cDNA prepared from A1 adult wasp ovaries with Superscript III (Invitrogen) as the template and the MegaScript RNAi Kit (Ambion). Resulting 300–400 bp dsRNA products or ds-*egfp* (control) were individually injected into the abdomen of day 1 *V. canescens* pupae (P1) at a volume of 0.5–1 µL with dsRNA concentration adjusted to 400–500 ng per µL. For dsRNA cocktails targeting multiple genes from a gene family, dsRNAs were mixed together in equal concentrations and injected as above. For the first knockdown experiment, expression of all gene family members was measured with qPCR (Fig. S14). For all subsequent experiments using dsRNA cocktails, only a single targeted gene was used to verify the knockdown efficacy. After emerging as adults (A1), the paired ovaries in treated females were explanted in phosphate-buffered saline (PBS) by dissection. One ovary was removed for total RNA isolation using the QuickRNA Mini-prep kit (Zymo) to assess knockdown of the targeted gene. Across all experiments performed in this study, the amount of total RNA isolated from each ovary was not significantly different between control and knockdown ovaries, indicating consistent RNA isolation efficiency (Fig. S15). Total RNA was synthesized into cDNA, which was used as the template in reverse transcriptase (RT) quantitative (q) PCR assays that used primers corresponding to 70–160 bp regions of each targeted gene (Table S1). Within an experiment, an internally consistent amount of total RNA was used for each cDNA reaction. Each PCR product was cloned into pSC-A-amp/kan that was Sanger-sequenced to confirm the identity and used to generate an absolute standard curve by serially diluting the plasmid (10² to 10^7^ copies) using the Rotor-Gene SYBR Green PCR kit and specific qPCR primers (Table S1). Copy number of each transcript from treatment samples was determined by fitting the RT-qPCR data to the standard curve. For each gene, at least two females were examined (i.e., two biological replicates) while each RT-qPCR assay was run in quadruplicate (four technical replicates). For statistical analysis, the copy number of each gene was compared between females that were injected with ds-RNA to the target gene versus females that were injected with *egfp* (control) by first log-transforming the data to normalize and then analyzing using a two-tailed unpaired *t*-test. Statistical analyses were performed using R v4.4.2 with a *P*-value ≤ 0.05 considered significant.

### Transmission electron microscopy (TEM)

Two types of samples were processed for TEM: (i) ovaries from pupae (P4, P6, and P8) and newly emerged adult females (A1), and (ii) ovaries from A1 wasps injected with dsRNA as P1 pupae. All ovaries used for TEM were fixed in 3% glutaraldehyde in 100 mM Sorenson’s Phosphate Buffer (pH 7.4) at 4°C for 1 week. They were then rinsed 3 times for 15 min with 100 mM Sorenson’s Phosphate Buffer containing 300 mM sucrose. Samples were post-fixed in 1% osmium tetroxide in 100 mM Sorenson’s Phosphate Buffer for 1 h. Samples were then washed three times for 10 min in DI water followed by dehydration using graded ethanol solutions in DI water (30%, 50%, 75%, two 95%, and two 100% ethanol) each for 15 minutes. The samples were further dehydrated with two 10 minute 99.5% acetone washes and two 15 minute propylene oxide washes. Epoxy (a mixture of Epon 812: Araldite: DDSA: DMP-30 mixed by weight at 1:1:2.044:0.0932) was gradually infiltrated into the sample using the following mixes of polypropylene : epoxy for 2 hours each: 2:1, 1:1, 1:2, and finally two 2 hour washes of epoxy. Samples were embedded in epoxy and dried overnight in an oven at 65°C. Ovaries were thin-sectioned using an ultramicrotome, mounted on copper grids, and post-stained with uranyl acetate and lead citrate. Samples were then examined using a JEM-1011 Transmission Electron Microscope, equipped with an XR80M Wide-Angle Multi-Discipline Mid-Mount CCD Camera from AMT (Advanced Microscopy Techniques).

### Scanning electron microscopy (SEM)

*V. canescens* eggs were dissected from the lateral oviducts of A1 adult females in PBS that were either untreated or injected with different dsRNAs as P1 pupae. Eggs were fixed in 3% glutaraldehyde in 100 mM Sorenson’s Phosphate Buffer (pH 7.4) for 2 days at 4°C followed by rinsing two times for 10 min in 100 mM Sorenson’s Phosphate Buffer containing 300 mM sucrose. Eggs were then placed on coverslips precoated with poly-L-lysine (Electron Microscopy Sciences) overnight in 100 mM Sorenson’s Phosphate Buffer containing 300 mM sucrose. Samples were post-fixed in 1% osmium tetroxide in 100 mM Sorenson’s Phosphate Buffer for 1 h, washed three times for 10 min in DI water followed by dehydration using graded ethanol solutions in DI water (30%, 50%, 75%, two 95%, and two 100% ethanol). Samples were then washed twice for 15 min in 100% ethanol and hexamethyldisilazane (HMDS) in ratios of 2:1, 1:1, and 1:2, followed by two 15 min washes in 100% HMDS and air-drying overnight. Eggs were coated with a mixture of gold and palladium to 30 nm thickness using a Leica EM ACE600 sputter coater. Samples were then examined using a Thermo Fisher Scientific Teneo Field Emission-Scanning Electron Microscope.

### Light microscopy

Eggs collected from the lateral oviducts as described for scanning electron microscopy and ovaries were also examined unfixed by placement in PBS on slides using a Keyence VHX-E100 digital microscope with phase-contrast optics. Debris was removed from the background of the ovary image using Photoshop.

### Encapsulation assays

Females injected with dsRNAs as P1 pupae were maintained until adult emergence. Three to four females for each ds-RNA that knocked a given nudivirus gene were placed individually in a small container to which *E. kuehniella* fifth instars were added sequentially. Three to four females treated with ds-*egfp* served as a negative control. Larvae observed to be parasitized in each container were transferred to another container with medium and held under conditions used for rearing for 48 h. Each larva was then dissected in PBS to isolate the *V. canescens* egg that was present. Each wasp egg was then examined by light microscopy, as described above, and scored as unencapsulated if no hemocytes were bound to its surface or encapsulated if bound hemocytes were present over all or a portion of the egg. The percentage of eggs that were encapsulated for each knockdown treatment was then compared to the percentage of eggs that were encapsulated from females that were treated with ds-*egfp* by a Fisher’s exact test using JMP 16.0.

## Data Availability

All relevant data are available within the article and its supplemental material.

## SUPPLEMENTAL MATERIAL

Supplemental material jvi.01305-25-s0001.pdf Table S1; Fig. S1 to S16.

ASM does not own the copyrights to Supplemental Material that may be linked to, or accessed through, an article. The authors have granted ASM a non-exclusive, world-wide license to publish the Supplemental Material files. Please contact the corresponding author directly for reuse.
