# Supplementary material for: Four nudivirus core genes present in the genome of Venturia canescens are required for virus-like particle formation and prevention of encapsulation of parasitoid wasp eggs
Source: J Virol. 2025 Nov 20;99(12):e01305-25. doi: 10.1128/jvi.01305-25 (PMC12724227; doi:10.1128/jvi.01305-25)
Supplement: Supplemental material — Table S1; Fig. S1 to S16. [file jvi.01305-25-s0001.pdf]

Table S1 Primers used in the study.

Primer purpose	Primer name	Primer sequence 5' – 3'
qPCR	*OrNVorf18-like-L*	AGATGCTGCAAACGATTGGC
	OrNVorf18-like-R	GAAGTCTTCGGCATTGAACGG
	OrNVorf41-1-like-L	ATCACCACTCATGCGGGTTG
	OrNVorf41-1-like-R	TTTGCCGGATGAATGCTTGC
	OrNVorf41-2-like-L	GATTACTTGGTGCCAGTTCACG
	OrNVorf41-2-like-R	TTGTAGTTCCGCGGGAGATG
	OrNVorf41-3-like-L	AGTGGTAGTTGGATTGGCTTCC
	OrNVorf41-3-like-R	TTGAGCAGACACGTCCGAAC
	OrNVorf41-4-like-L	AATTAGCGCGGTGGTTCTTG
	OrNVorf41-4-like-R	CAAAGCCGAAGACCCTTAGTTG
	OrNVorf41-5-like-L	GGAAATTCCTCCCGATTGCAAC
	OrNVorf41-5-like-R	AGATCAGTGCGGCTAGAATCATC
	OrNVorf41-6-like-L	AAGAAGGTTACTCCCCAGTGC
	OrNVorf41-6-like-R	AACAAGCAGAGCGCAGTAAG
	OrNVorf47-1-like-L	ATGAAAAAGGAGATGGACGAAGAGA
	OrNVorf47-1-like-R	TCGTCGCGCATGATATAAAATTTGA
	OrNVorf47-2-like-L	TGAAAACCTCGCTGAAAGACTTTAC
	OrNVorf47-2-like-R	GTGCCACCTTGAAACCAAACTAATA
	OrNVorf47-3-like-L	TGTACCAGACGGAATTCGATAGTAC
	OrNVorf47-3-like-R	TCAATCCGTAGCACGAGTATAGTTT
	OrNVorf61-like-L	CGTCCATGGGCTACAAAACAC
	OrNVorf61-like-R	TTTCGTTGACTTCGCTGGTG
	OrNVorf76-like-L	GTTAATGCGTCGCCCTCTTATG
	OrNVorf76-like-R	ATCGCCGCGATGTATAAATTTTC
RNAi	*OrNVorf18-like-RNAi-L*	TAATACGACTCACTATAGGGATCGAAGATGACGAGTTGCTGT
	OrNVorf18-like-RNAi-R	TAATACGACTCACTATAGGGGTCGCTCAAATTGTCGATAGGC
	OrNVorf41-1-like-RNAi_L	TAATACGACTCACTATAGGGTTGCTTTCCGACCGTTTACTCT
	OrNVorf41-1-like-RNAi_R	TAATACGACTCACTATAGGGCGGGTCCTGACAAAATGGTCAT
	OrNVorf41-2-like-RNAi_L	TAATACGACTCACTATAGGGTGCTGCAACAATTTACAACATCG
	OrNVorf41-2-like-RNAi_R	TAATACGACTCACTATAGGGGCTCTTGGATATCAAATCGCTCT
	OrNVorf41-3-like-RNAi_L	TAATACGACTCACTATAGGGAGACCGAAATCAAATTCTTTGGCT
	OrNVorf41-3-like-RNAi_R	TAATACGACTCACTATAGGGTTTCGTTTCTTTGTCTTCAGGGC
	OrNVorf41-4-like-RNAi_L	TAATACGACTCACTATAGGGAGAACAAGGAAATGTACCCCCG
	OrNVorf41-4-like-RNAi_R	TAATACGACTCACTATAGGGGCCGTCGAAATCCAATGCATTA
	OrNVorf41-5-like-RNAi_L	TAATACGACTCACTATAGGGTCGAGTTCGATAACGCTAAGGA
	OrNVorf41-5-like-RNAi_R	TAATACGACTCACTATAGGGTCGGCTGTTTCTTGGAATTGC
	OrNVorf41-6-like-RNAi_L	TAATACGACTCACTATAGGGGGACCACGCAATGAGAAAGT
	OrNVorf41-6-like-RNAi_R	TAATACGACTCACTATAGGGTTAAGGCATTCTTTCCATCCA
	OrNVorf47-1-like-RNAi_L	TAATACGACTCACTATAGGGCAATACGATCCTTTCCGTTCTGTTG
	OrNVorf47-1-like-RNAi_R	TAATACGACTCACTATAGGGTATGAAGAACGAATCGGCAAGTCAA
	OrNVorf47-2-like-RNAi_L	TAATACGACTCACTATAGGGGACGAACAAGATACTCGAGCAATTT
	OrNVorf47-2-like-RNAi_R	TAATACGACTCACTATAGGGTGTATAGAAATTCTGAGGTCGCACT
	OrNVorf47-3-like-RNAi_L	TAATACGACTCACTATAGGGTGCTATTCATAATTTCACCTGCACC
	OrNVorf47-3-like-RNAi_R	TAATACGACTCACTATAGGGTACTCATTGTACCCATGGAATTGGT
	OrNVorf61-like-RNAi-L	TAATACGACTCACTATAGGGTTCAGGAAGTTCAACGACTCG
	OrNVorf61-like-RNAi-R	TAATACGACTCACTATAGGGCACCGTGAATCCCAATAAAACG
	OrNVorf76-like-RNAi-L	TAATACGACTCACTATAGGGGCTTTTTATGCTTGCCCTATTGG
	OrNVorf76-like-RNAi-R	TAATACGACTCACTATAGGGTGTTTTTGATATACTTGCATTTCTCGA
	EGFP_RNAiF	TAATACGACTCACTATAGGGCGAATTCACTAGTGATTTTACTTG
	EGFP_RNAiR	TAATACGACTCACTATAGGGGCGGGAATTCGATTTGACC

>OrNVorf18-like | No transmembrane domains
MDSNEAFTRAALYQSCKRSKILNNIDIENVDIQTLRALVNQHNAVSVRELPILISKYVVKFGLSFVVERFPKMRGYMN
PENIVVQIEDDELLYLQTHEGRAQMNIYTIVMKNIAVSVLGSSIASLREQLNSNSRVTEYPKIKVKSVAQPIPQQVVP
KTYTNKFDDVSFSPMLTSPLQRAVQSSKAGSSERDTNIHKHSDQVVNGRIIRRNELQAIRRMHRDRLTSRLRPIDNLS
DSAESVDNEDEVDDIEETEIERRSLLGRRSSISKFNDSTILLDDINDADEKYLEYTSPLSQSPRQDAANDWLNLDNVE
HQRHYATIEKTKIRSMPKTSSDTLIFPSESKQTINESEIIHKLADDGEVKTHELKTVDRQMDLEQQQKEDDEDDIISI
IEPSDVNKTTNGPDEAGNTREEFTDKSAPIVSDVSIDAEKHKAASTSDTDDKRITISFSDDEDD

>OrNVorf41-like-1 | 2 transmembrane domains
MASKKLLSDRLLSVIVPACTDLGNSIKFSTMTLIVMIGIALVLFIIMALYNWVRDVHAVNYNDQVEREKEHKNIMTIL
SGPATILVAALIIGITTHAGWHSSSTKRLAPLCSGLRQAFIRQTVAASNQ

>OrNVorf41-like-2 | 2 transmembrane domains
MGLLGASSRLQWMVSSDCSHLPRNYKVSSIIVILLAFITLVVMLAATIYNIVNTSQLGGSPDSFNSKYKDDVEDVQKA
LSYLSIASVVLTTLTLAALLWKYSAASKMAKKCLQSPPAALERFDIQEPIPWNKELFNESQ

>OrNVorf41-like-3 | 2 transmembrane domains
MSVRPKSNSLAARSRVNSTITPECSHLGKSIKISSTFVIVMIAIGMILMIAALIINSEDDGGRPEDKERKLNGVTITS
AVVVGLASLAAIWQFSVAARTVRTCLLKDETM

>OrNVorf41-like-4 | 2 transmembrane domains
MISQRKYNAKVSKEQRNVPPETPPNCDNLGRNIKLFSLSVIMLMIIVLIVLIIAMVMNNALDFDGEDKKQSILETLTI
ISAVVLGVAWITAIWHISVATKGLRLCQQLKK

>OrNVorf41-like-5 | 2 transmembrane domains
MSSSSGVSSEIPPDCNTRVKSFKLYGFIVIMMILAALILTIVTVVTVDTVEFDNAKEADRKTSMINGPSIGGAVLVIM
ACGIAIWQFQETAEITRLCMH

>OrNVorf41-like-6 | 2 transmembrane domains
MPDEKSTKSANEKFIKKVTPQCWNLAKWVKISGYMVIIFTVLTALCLFVAIIMDHAMRKCTEEEKSDIPRILVGVSFG
FIIATCLLGIWQYTVGAKTTRVCIIPPWMERMP

>OrNVorf47-like-1 | No transmembrane domains
MTNSSLFYVNTILSVLLVLLLAIFIVVPAPLKASFDRYSIDNNYDPATNAEVKYLYTSYKKPDNKKLLVWFQAGAFVM
KNRKTPYGLLNCLNDALVEFDILTFDLPIRSSYTVRDAMLTTNELLGRFKKYDEYYAAGFSTGALLMGSFMKKEMDEE
TSRKMGVPAIGIRFKAIVGLCGLYSTKFDDSNLESLFKFYIMRDDSNSHLYSCSDLDVPMLVIGATSEFLYTDQTRKF
HCAGKCEKKLFSETPLPHEFPLMMDLPEAKESAETVIQFLREN

>OrNVorf47-like-2 | No transmembrane domains
MTLWYFVNIILTLILIILSVVFIIIPAPMKTSLKDFTLVASESDPATNAEVKDLYTLYSRPNNKKLLVWFQGGAFLLN
NRKTSYGILNALNDALIEFDVLVFDYPLRFSHSVHDAMLATNKILEQFYAYDEYYAAGFSVGALLMGAFMQKELNKEI
ARKVDVPVIGIQFKAIISLCGVYSMKFDCLLLNLISRLYIMRGTPNPHLYSYHGLLSVPLLVISATSEFLYTETYKFT
KSTACELKVFRGALLPHEFPLMLEFPETKESIESVIEFLRAN

>OrNVorf47-like-3 | 1 transmembrane domain
MTLWYFVNTILSLLCVILIVLFIISPAPLKKSMEQYILAGESDPATNTKVEDLYTLYSRPNNKKLFVWFQGGAFLLNN
RKTSYGILNALNDALIEFDILVFDYPLRFSYSVHDAMLATNKILDQFHGYNEYYAAGYSAGALLMGAFMQKELNKEIA
RKMDVPVIGIQFKAIIGICGLYQTEFDSTLLTSLFQFYIMRGTPNKKLYSCYGLSVPTLVIGATAEFLYTQTKKFTKS
TGCKLKVFQDTSLPHEFPLMLEFPETKESVESMIEFLRAN

>OrNVorf61-like | 2 transmembrane domains
MNANKITELDGASMGYKTPQQSFQEALNFTSEVNEIDVDSKKEISLLELFRKFNDSVITYPVVVLLINTFSVLIIIFQ
RSLALIIKLIVLVLYVVLLGFTVFTFKNRN

>OrNVorf76-like | 1 transmembrane domain
MLFMLALLVFVLTLCGLFFAKDSFINREMQVYQKQLQEPWLMRRPLMAYNGTRRLTLKRKFIHRGDPKKV

Fig S1 Fasta formatted protein sequences showing predicted transmembrane domains.

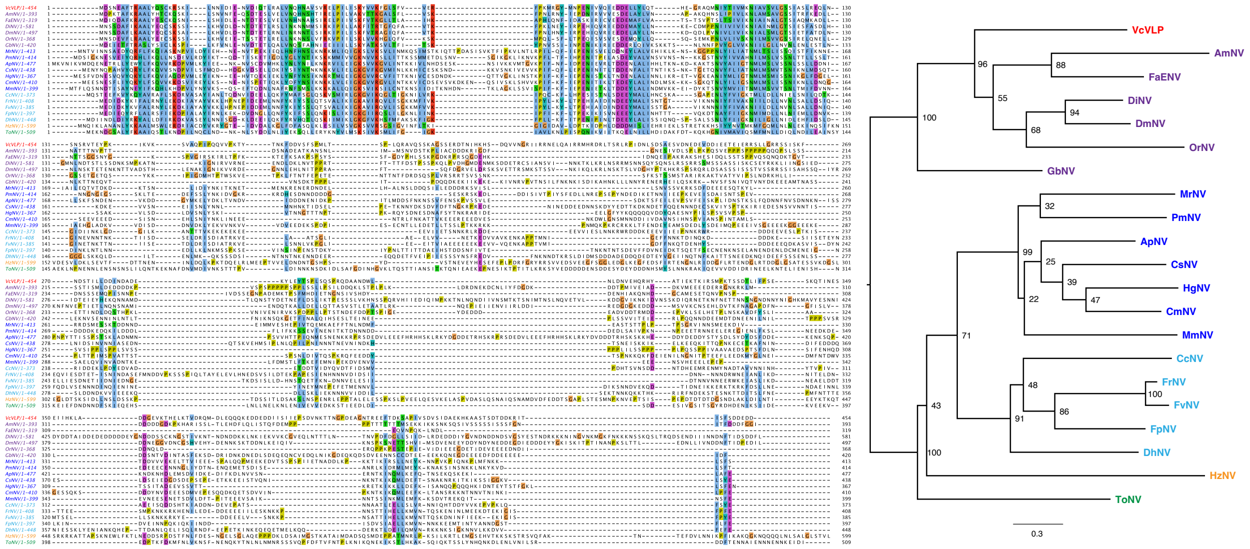

Fig S2 Conservation of domains and evolutionary relatedness of OrNVorf18-like proteins from *V. canescens* and select nudiviruses. Colors in the alignment highlight conserved residues and amino acid residue characteristics. Numbers indicate relative positions of aligned residues in each protein. Taxa include Apis mellifera nudivirus (AmNV), Aratus pisonii nudivirus (ApNV), Callinectes sapidus nudivirus (CsNV), Carcinus maenas nudivirus (CmNV), Crangon crangon nudivirus (CcNV), Dikerogammarus haemobaphes nudivirus (DhNV), Drosophila innubila nudivirus (DiNV), Drosophila melanogaster nudivirus (DmNV), Faxonius propinquus nudivirus (FpNV), Faxonius rusticus nudivirus (FrNV), Faxonius virilis nudivirus (FvNV), Fopius arisanus endogenous nudivirus (FaENV), Gryllus bimaculatus nudivirus (GbNV), Heliothis zea nudivirus 1 (HzNV), Homarus gammarus nudivirus (HgNV), Macrobrachium rosenbergii nudivirus (MrNV), Menippe mercenaria nudivirus (MmNV), Oryctes rhinoceros nudivirus (OrNV), Penaeus monodon nudivirus (PmNV), Tipula oleracea nudivirus (ToNV), and Venturia canescens endogenous nudivirus (VcVLP). Taxa are colored to indicate nudiviruses in the genera *Alphanudivirus* (purple), *Betanudivirus* (orange), *Deltanudivirus* (blue), *Gammanudivirus* (light blue), and *V. canescens* VLP sequences (red). Accession numbers are given in Supplementary Table 2.

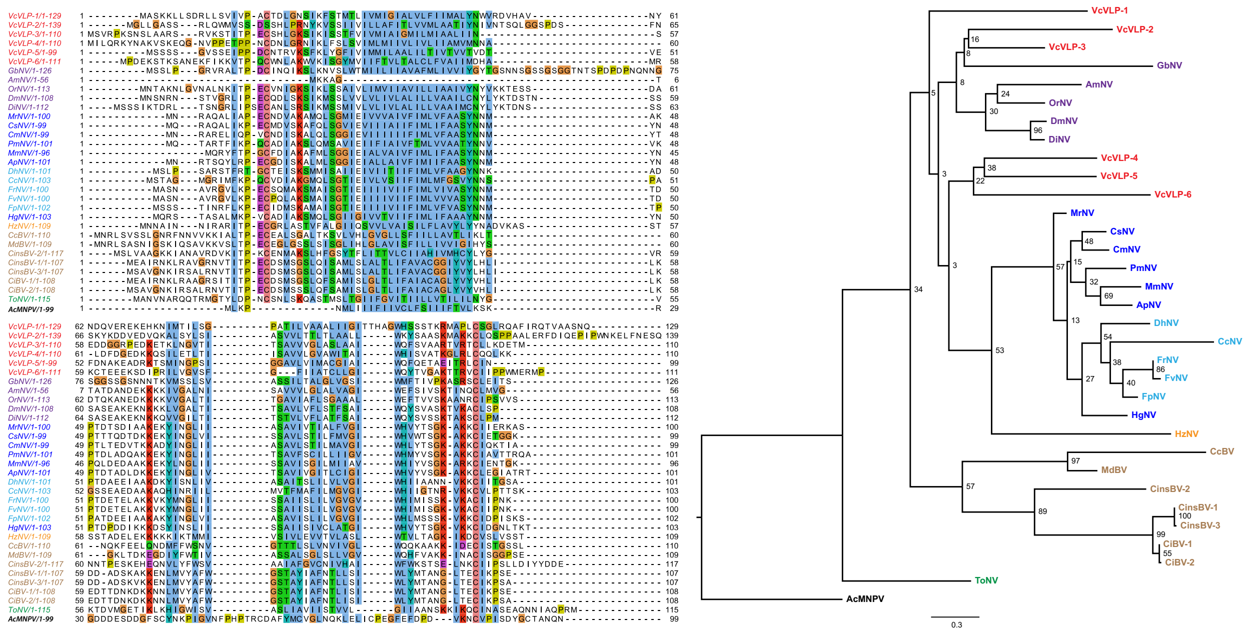

Fig S3 Conservation of domains and evolutionary relatedness of OrNVorf41-like proteins from *V. canescens* and select nudiviruses. Alignment characteristics, taxa names and coloration are as given in Fig S2. Additional taxa include Microplitis demolitor bracovirus (MdBV), Chelonus inanitus bracovirus (CiBV) and Chelonus insularis bracovirus (CinsBV).

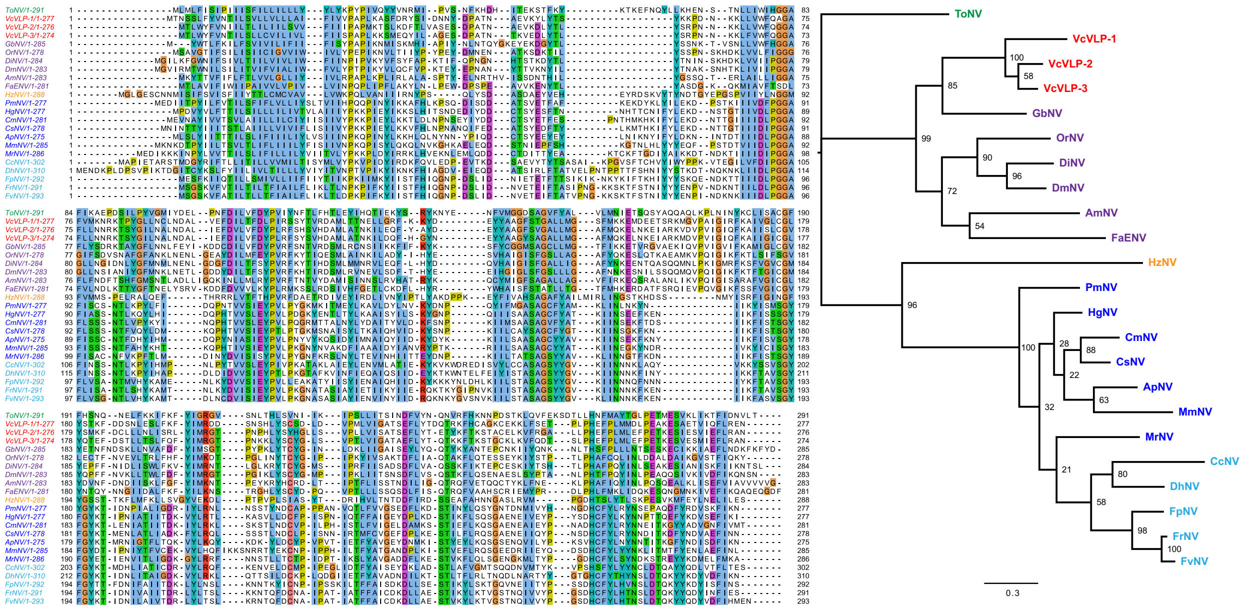

Fig S4 Conservation of domains and evolutionary relatedness of OrNVorf47-like proteins from *V. canescens* and select nudiviruses. Alignment characteristics, taxa names and coloration are as given in Fig. S2.

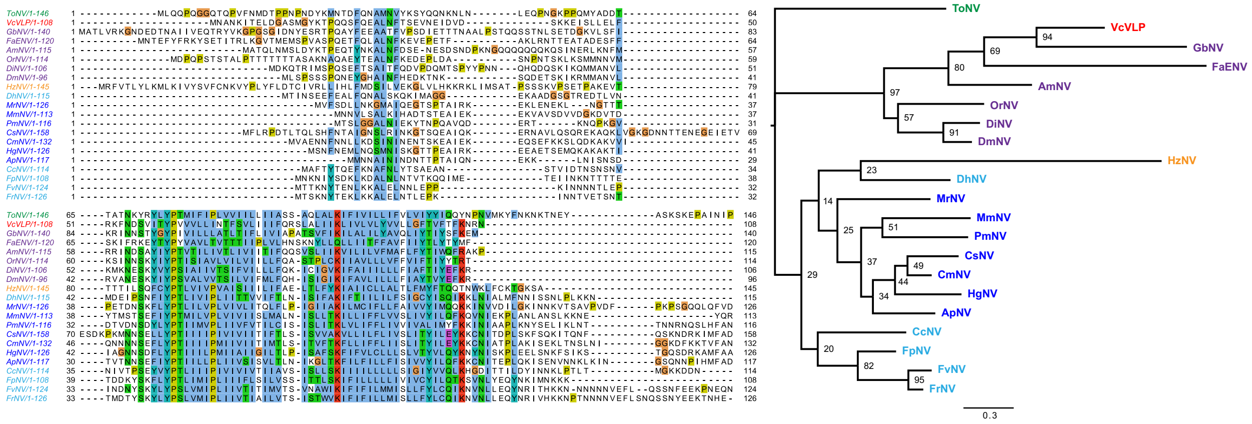

Fig S5 Conservation of domains and evolutionary relatedness of OrNVorf61-like proteins from *V. canescens* and select nudiviruses. Alignment characteristics, taxa names and coloration are as given in Fig. S2.

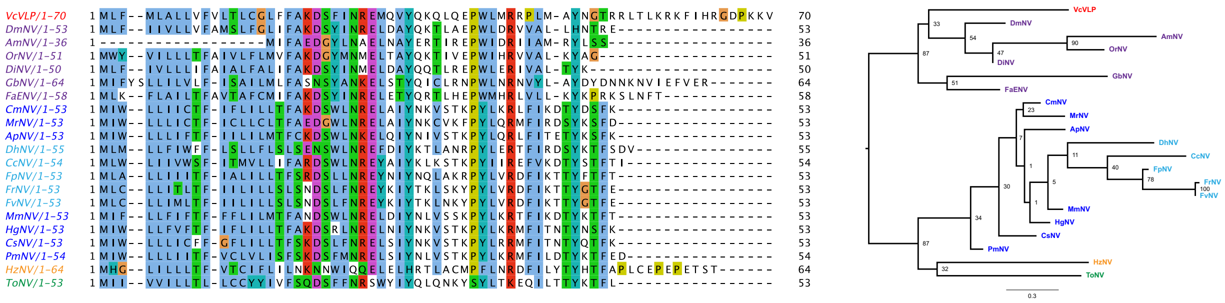

Fig S6 Conservation of domains and evolutionary relatedness of OrNVorf76-like proteins from *V. canescens* and select nudiviruses. Alignment characteristics, taxa names and coloration are as given as given in Fig. S2.

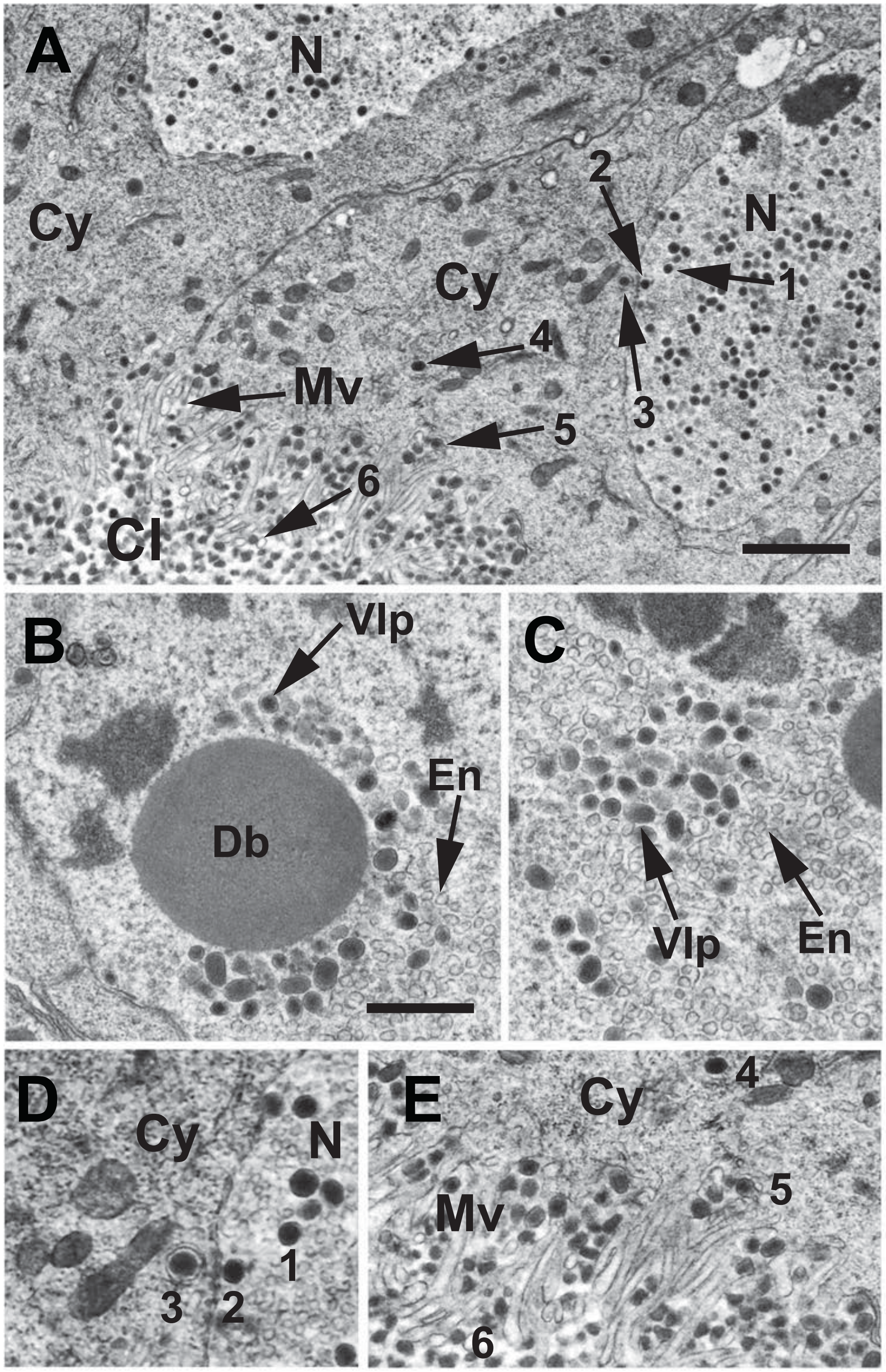

Fig S7 VcVLPs in calyx cells injected with ds-*egfp.* (A) Low magnification TEM image showing two calyx cells (Phase 3) from a newly emerged *V. canescens* adult (A1) that was injected with ds-*egfp* when a P1 pupa. VLPs are present in the nuclei (N) and cytoplasm (Cy) of each calyx

cell, which have plasma membranes forming microvillae (Mv) that extend into the calyx lumen (Cl). Numbers identify representative VLPs in: 1) the nucleus, 2) the nucleus near the nuclear membrane, 3) the cytoplasm near the nuclear membrane, 4) traversing the cytoplasm, 5) in a microvilla, and 6) in the calyx lumen. Scale bar = 1 μm. (B) Higher magnification TEM image showing VLP envelopes (En) and mature VLPs in proximity to a dense body (Db) in a calyx cell nucleus. Scale bar = 400 nm. (C). Higher magnification TEM image showing envelopes and mature VLPs in a calyx cell nucleus that are not in close proximity to a dense body. (D) Higher magnification TEM image of VLPs 1-3 shown in A. Note that VLP 1 and 2 have a single envelope, while VLP 3 has two envelopes from budding through the nuclear membrane. (E) Higher magnification TEM image of VLPs 4-6 in A. VLP 4 and 5 have no second envelope while VLP 6 in the calyx lumen also has only one membrane. C-E are at the same magnification as B.

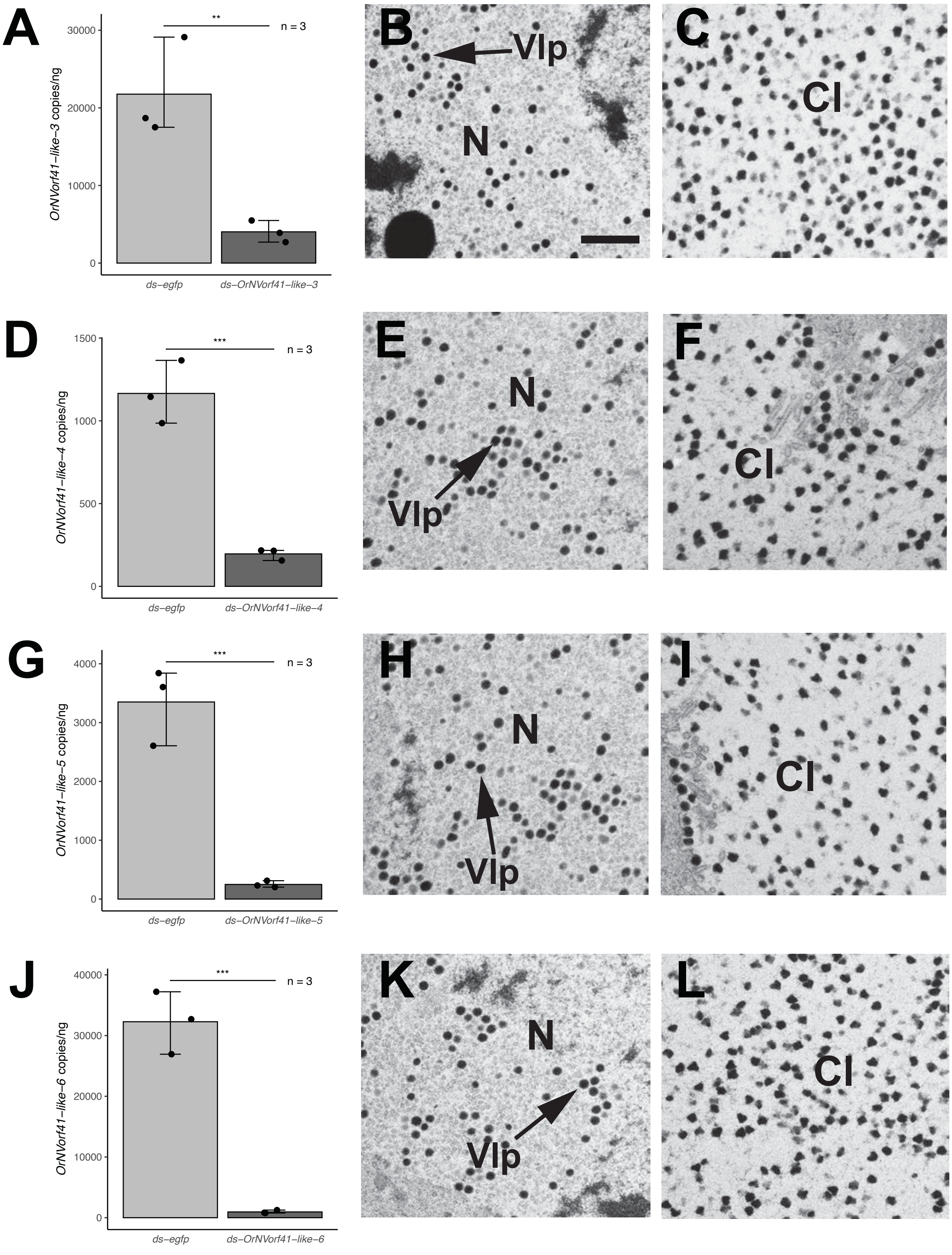
A
**
n = 3
30000
20000
10000
0
OrNVorf41-like-3 copies/ng
ds-egfp
ds-OrNVorf41-like-3
B
Vlp
N
C
Cl
D
***
n = 3
1500
1000
500
0
OrNVorf41-like-4 copies/ng
ds-egfp
ds-OrNVorf41-like-4
E
N
Vlp
F
Cl
G
***
n = 3
4000
3000
2000
1000
0
OrNVorf41-like-5 copies/ng
ds-egfp
ds-OrNVorf41-like-5
H
N
Vlp
I
Cl
J
***
n = 3
40000
30000
20000
10000
0
OrNVorf41-like-6 copies/ng
ds-egfp
ds-OrNVorf41-like-6
K
N
Vlp
L
Cl

Fig S8 RNAi knockdown of *OrNVorf41-like-3-6* causes no defects in VLP formation. (A) Transcript abundance of *OrNVorf41-like-3* per ng of total RNA in ovaries from A1 females injected with ds-*egfp* or ds-*OrNVorf41-like-3* or as P1 pupae. Bars show mean values ± 1 standard error. Sample sizes for each treatment are indicated to the right while asterisks indicate significance (two-tailed t-test, ** p<0.01). (B) TEM image of a calyx cell nucleus (N) from a female treated with ds-*OrNVorf41-like-3* containing morphologically normal VLP envelopes and mature VLPs (VLP). Scale bar = 1 μm. (C) TEM image of the calyx lumen (Cl) from a female treated with ds-*OrNVorf41-like-3* containing morphologically normal mature VLPs. (D-F, G-H, J-L) Knockdown data, TEM image of a calyx cell nucleus and TEM image of the calyx lumen for females treated with ds-*OrNVorf41-like-4, -5,* or *-6* respectively, with details as indicated for images A-C. Results in D, G, and J indicate each gene was significantly knocked down (two-tailed t-test, *** p<0.001) while TEM analysis detected no alterations in VLP morphology in calyx cell nuclei or the calyx lumen. The images shown in C, F, I and L show different densities of mature VLPs in the calyx lumen but this reflected local variation across the lumen as a whole. Images in C, E, F, H, I, K, and L at the same magnification as B.

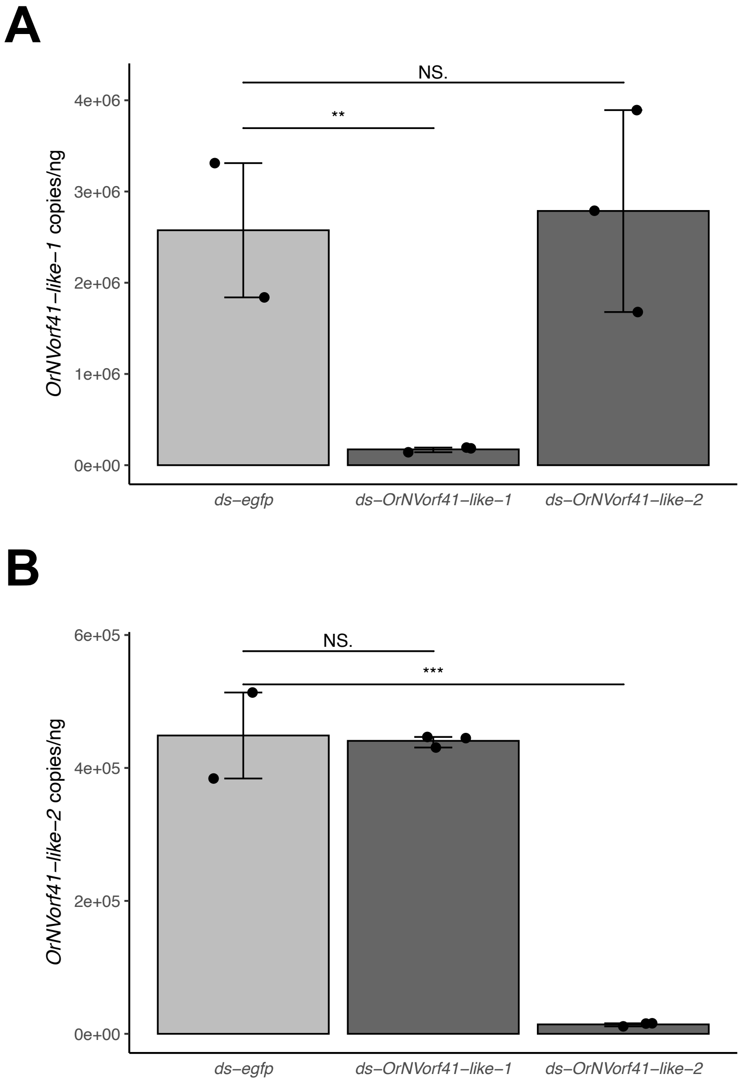

Fig S9 ds-*OrNVorf41-like-1* and ds*OrNVorf41-like-2* do not exhibit off-target effects. (A) Transcript abundance of *OrNVorf41-like-1* per ng of total RNA in ovaries from A1 females that were treated with ds-*egfp,* ds-*OrNVorf41-like-1* or *ds-OrNVorf41-like-2* as P1 pupae. (B) Transcript abundance of *OrNVorf41-like-2* per ng of total RNA in ovaries from A1 females that were treated with ds-*egfp,* ds-*OrNVorf41-like-1* or *ds-OrNVorf41-like-2* as P1 pupae. N =2 for the ds-*egfp* treatments while N = 3 for the ds-*OrNVorf41-like-1* or *ds-OrNVorf41-like-2* treatments. Asterisks indicate significance when the ds-*OrNVorf41-like-1* or *ds-OrNVorf41-like-2* treatments were compared to the *egfp* control (two-tailed t-test, ** p<0.01, *** p<0.001), while NS indicates no significant difference.

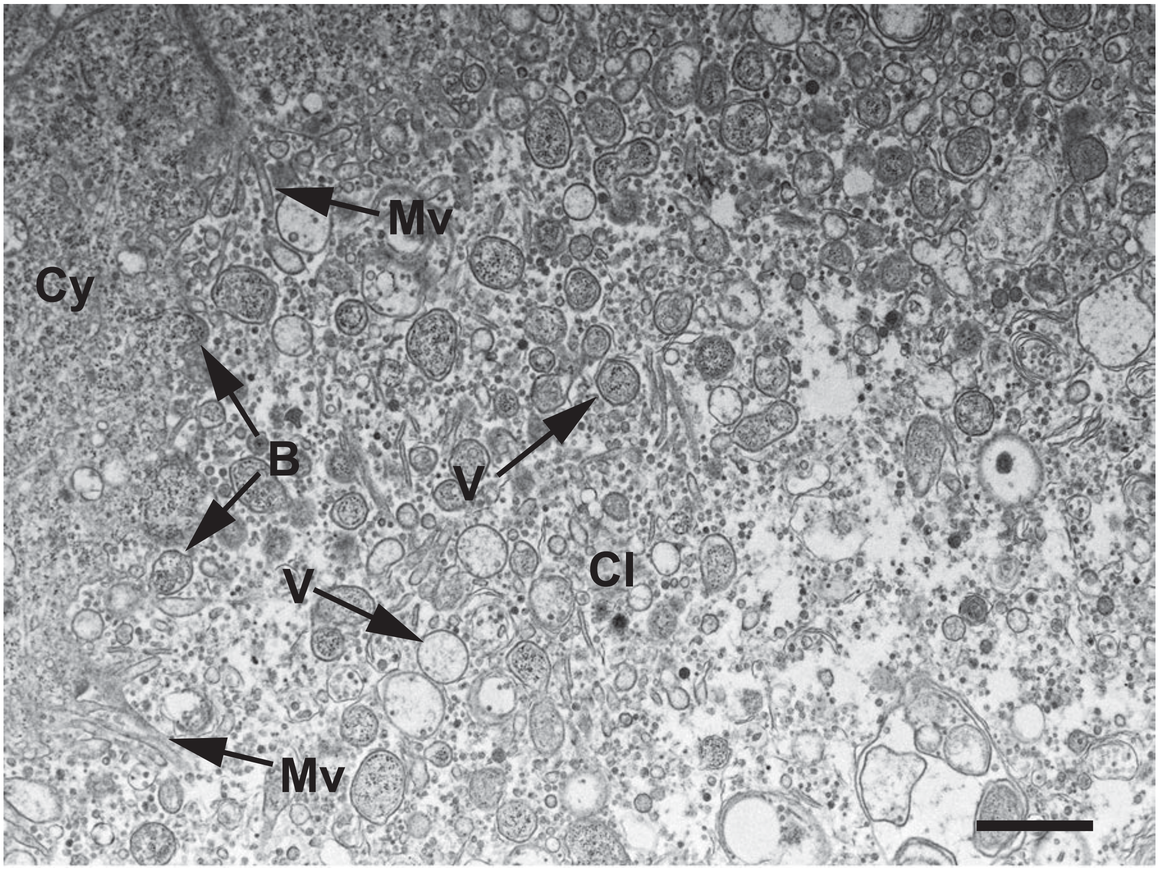

Fig S10 TEM image showing the calyx cell (left) and the calyx lumen from an A1 female injected with ds-*OrNVorf18-like.* The cytoplasm (Cy) and plasma membrane with microvillae (Mv) is visible to the left. The surface of the plasma membrane is in the process of budding (B) which results in large numbers of vesicles of varying size in the calyx lumen. Some of these vesicles contain cytoplasm while other do not. Scale bar = 1 μm.

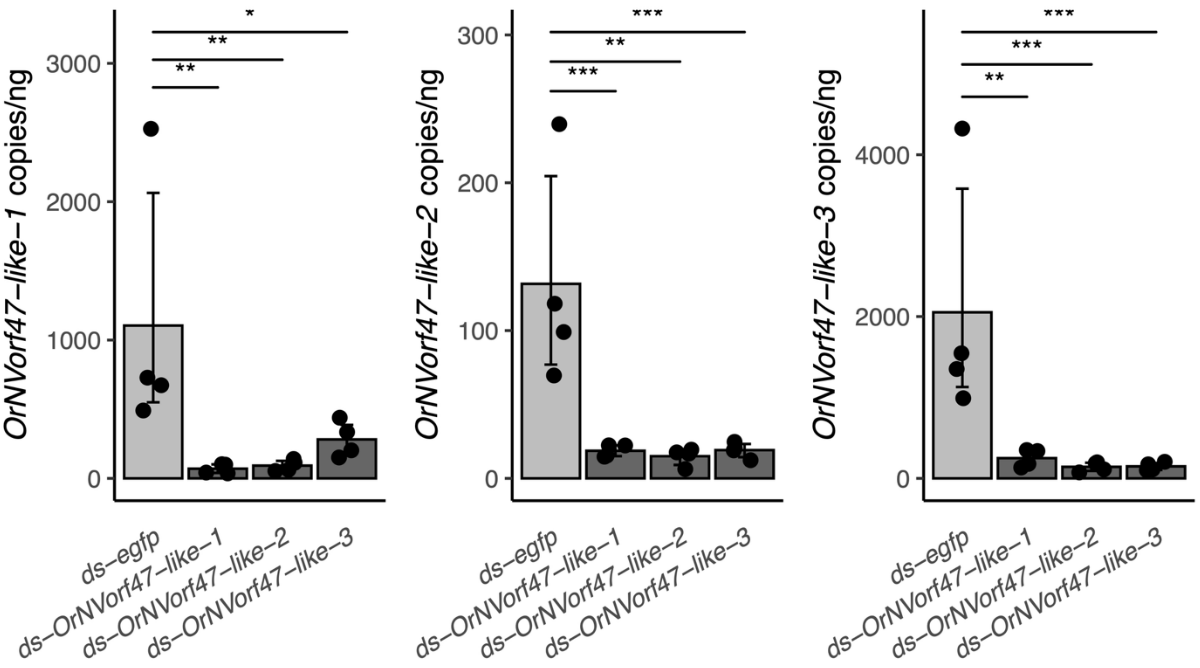

Fig S11 ds*OrNVorf47-like-1-3* exhibit off-target effects. (A) Transcript abundance of each *OrNVorf47-like* gene per ng of total RNA in ovaries from A1 females that were treated with ds-*OrNVorf47-like-1 ds-OrNVorf47-like-2, ds-OrNVorf47-like-3,* or ds-*egfp,* as P1 pupae. Each dsRNA designed to a given family member resulted in transcript abundances for *OrNVorf47-like-1, -2,* or *-3* that were significantly lower than from the ds-*egfp* control. N =4 for each treatment.

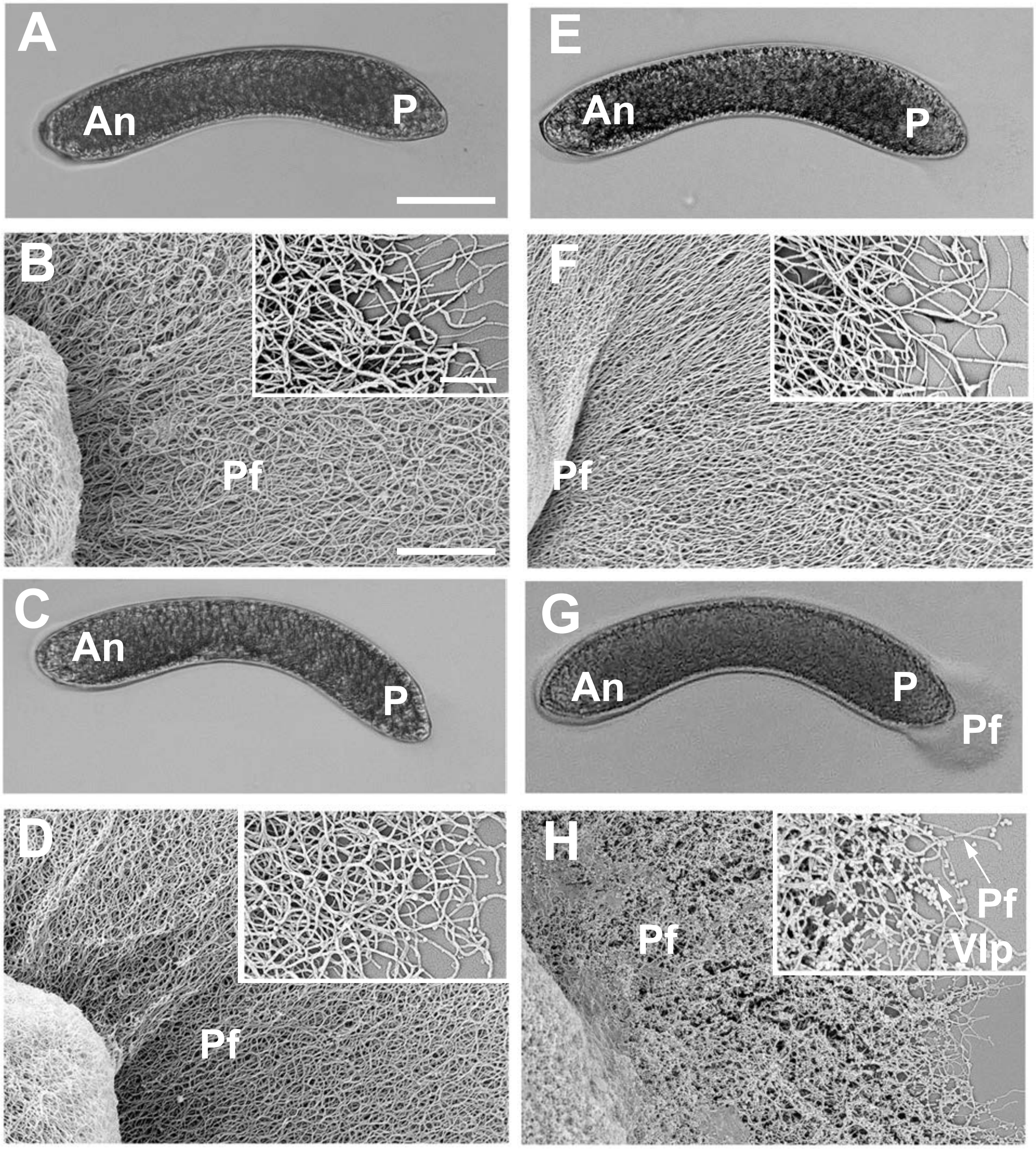

Fig S12 *V. canescens* eggs from A1 females injected with ds-*OrNVorf-like-18,* ds-*OrNVorf-like-41*-cocktail, ds-*OrNVorf-like-61,* or ds-*OrNVorf-like-76* as P1 pupae. Eggs were collected from reservoirs in the lateral oviducts after passage through the calyx lumen. (A) Light microscopy image of an egg from a ds-*OrNVorf-like-18* treated female with anterior (An) oriented to the right and no posterior filament visible at the posterior (P) end of the egg. (B) Low and high (insert)

SEM images of posterior filaments (Pf) showing almost no mature VLPs are attached. (C, D and E, F) Light and SEM images as defined in A and B of eggs from females treated with ds-*OrNVorf-like-41*-cocktail or ds-*OrNVorf-like-61.* Posterior filaments are not visible in the light microscopy images while SEM images show that almost no mature VLPs are attached to posterior filaments. (G, H) Light and SEM images as defined in A and B of eggs from females treated with ds-*OrNVorf-like-76.* Posterior filaments are visible in the light microscopy image while the SEM images show that large numbers of VLPs are attached to posterior filaments. Scale bar in A = 20 μm with images in C, E and G at the same magnification. Scale bar for the low magnification image in B = 10 μm while scale bar for the high magnification image = 5 μm with images in D, F and H at the same magnification.

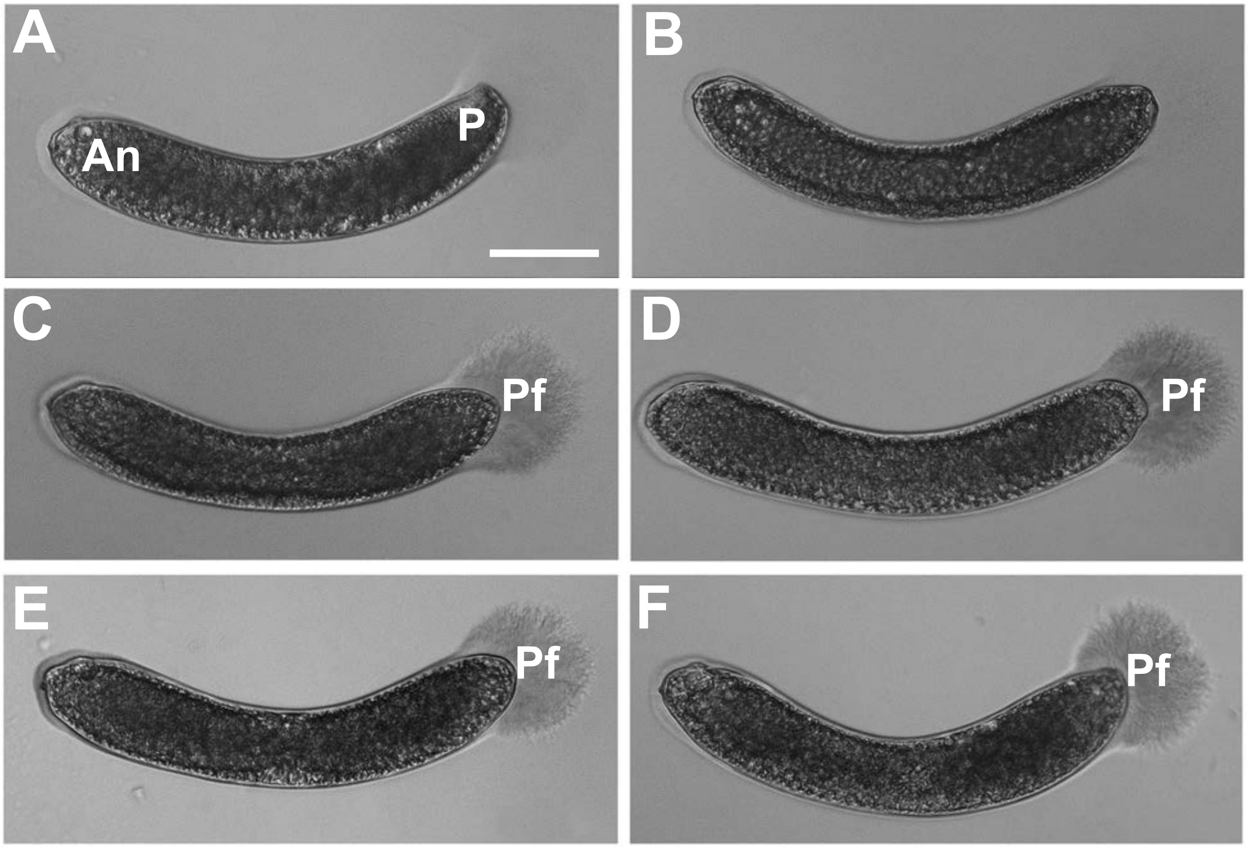

Fig S13 Light microscopy images of *V. canescens* eggs from A1 females injected with ds-*OrNVorf-41-like-1* (A), *-2* (B), *-3* (C), *-4* (D), *-5* (E), or *-6* (F). Eggs were collected from reservoirs in the lateral oviducts after passage through the calyx lumen. Each egg is oriented with anterior (An) to the left and posterior (P) to the right as labeled in A. The fan like structure formed by posterior filaments (Pf) is very weakly visible on eggs shown in A and B, but is readily visible on eggs shown in C-F. Scale bar in A = 20 μm with other images at the same magnification.

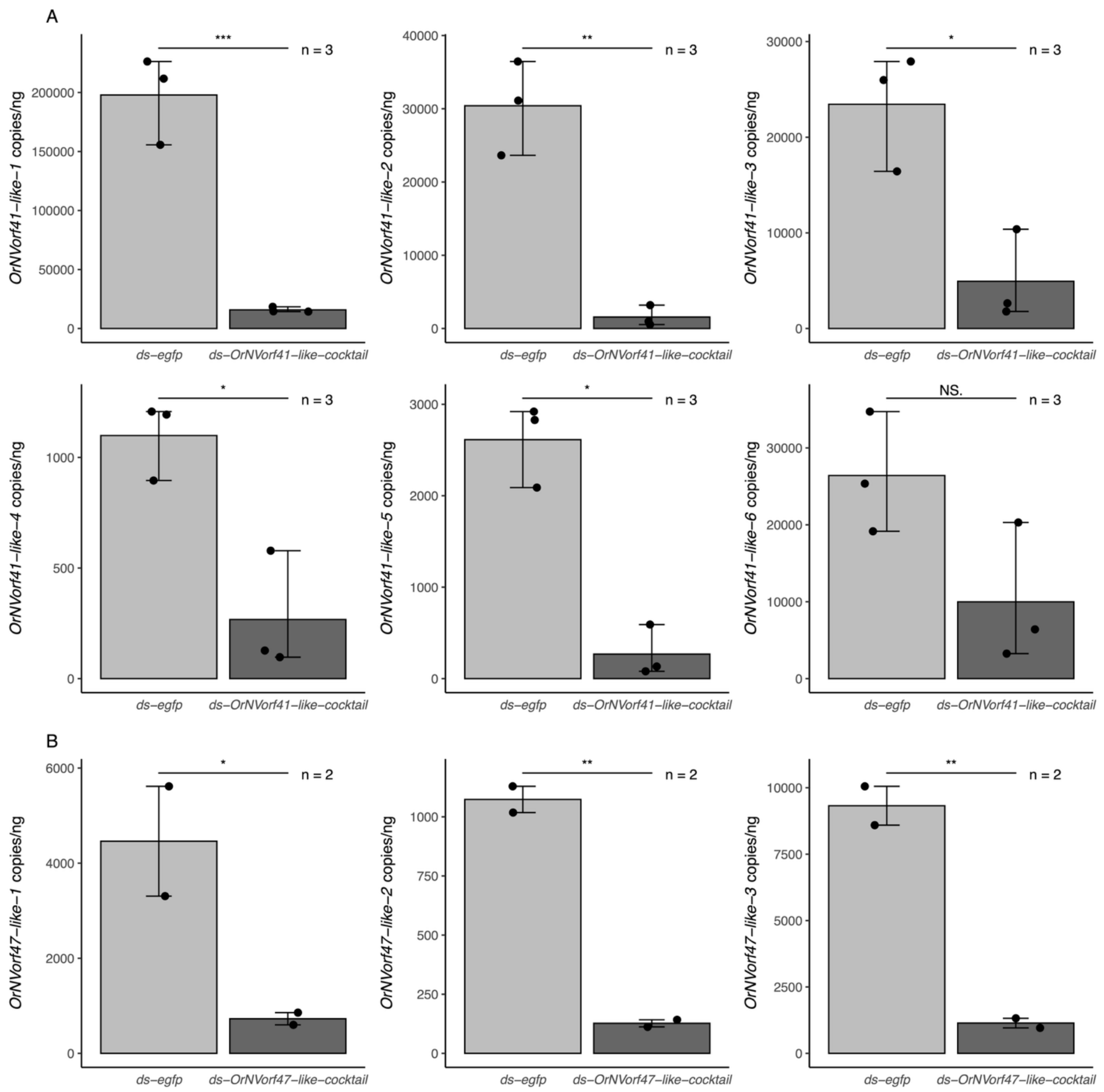

Fig S14 dsRNA cocktails are effective against all paralogs in *OrNVorf41-like* (A) and *OrNVorf47-like* (B) gene families. Note that the *ds-egfp* controls used for the *ds-OrNVorf41-like*-cocktail are the same as the controls used for the *ds-OrNVorf41-like-1* treatment shown in Figure 3D (three cohorts of wasps were injected with *ds-egfp, ds-OrNVorf41-like*-cocktail, and *ds-OrNVorf41-like-1* in the same experiment). One of the samples treated with the *ds-OrNVorf41-like*-cocktail was

an outlier compared to other knockdown samples for *OrNVorf41-like-6* expression, which resulted in a non-significant *p*-value for this gene.

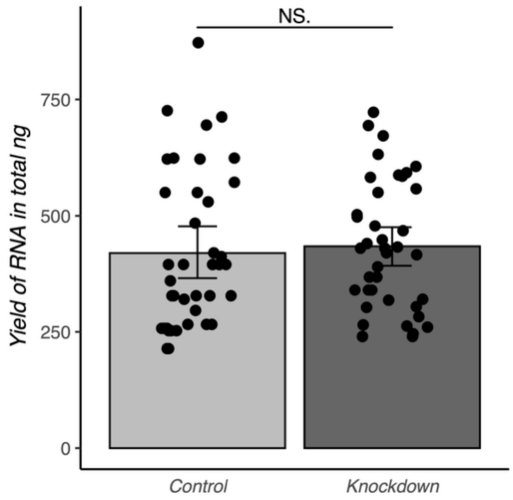

Fig S15 Yields of total RNA from ovary RNA extractions did not significantly differ across all samples used in this study.

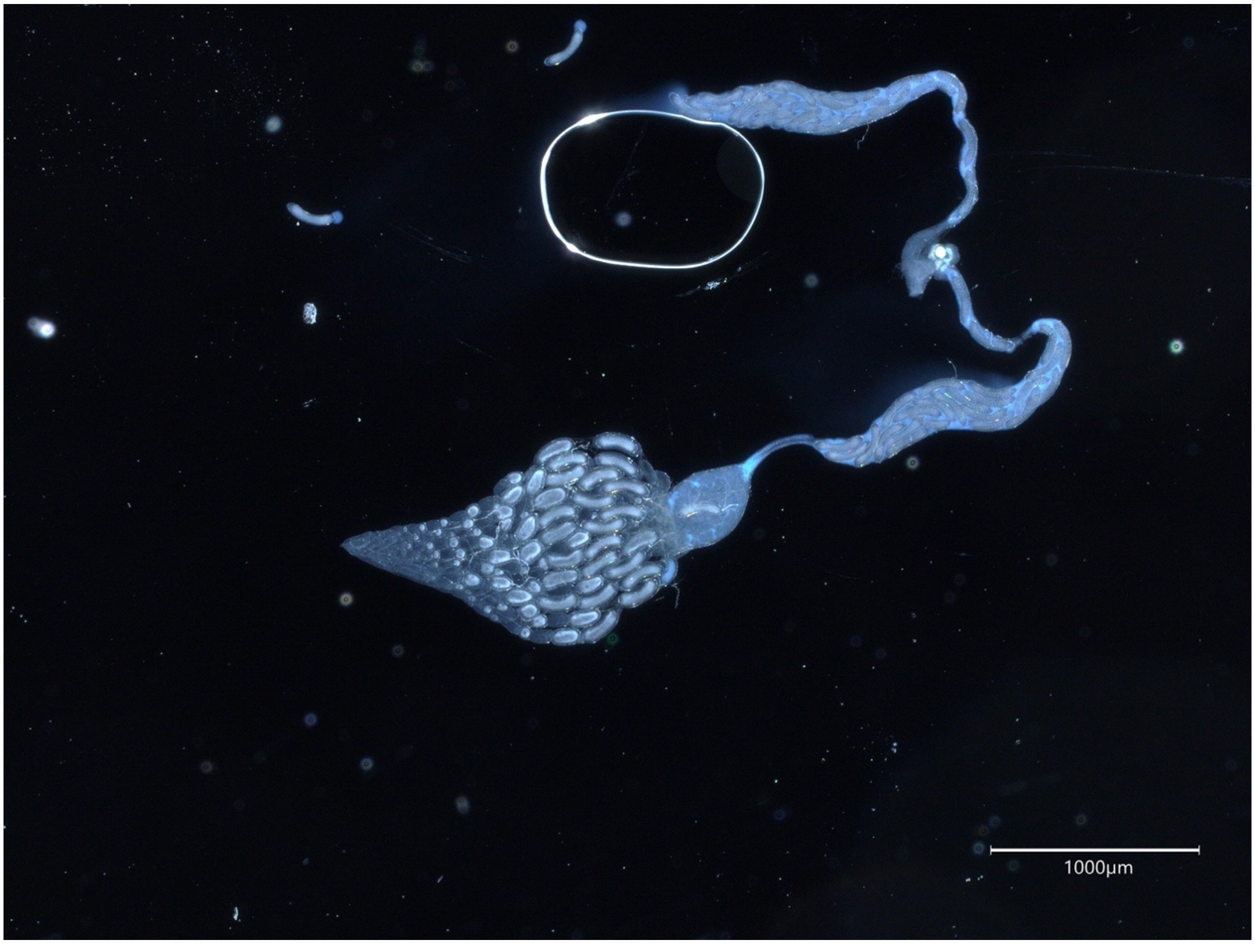

Fig S16 Original image shown in Figure 5A, without debris removal from the black background.
